# Toxoplasma DJ-1 Regulates Organelle Secretion by a Direct Interaction with Calcium-Dependent Protein Kinase 1

**DOI:** 10.1128/mBio.02189-16

**Published:** 2017-02-28

**Authors:** Matthew A. Child, Megan Garland, Ian Foe, Peter Madzelan, Moritz Treeck, Wouter A. van der Linden, Kristina Oresic Bender, Eranthie Weerapana, Mark A. Wilson, John C. Boothroyd, Michael L. Reese, Matthew Bogyo

**Affiliations:** a Department of Pathology, Stanford University School of Medicine, Stanford, California, USA; b Department of Life Sciences, Imperial College London, London, United Kingdom; c Department of Biochemistry, Redox Biology Center, University of Nebraska, Lincoln, Nebraska, USA; d Department of Microbiology and Immunology, Stanford University School of Medicine, Stanford, California, USA; e Department of Parasitology, National Institute for Medical Research, London, United Kingdom; f Department of Chemistry, Boston College, Chestnut Hill, Massachusetts, USA; g Department of Pharmacology, University of Texas Southwestern Medical Center, Dallas, Texas, USA; University of Pittsburgh

## Abstract

Human DJ-1 is a highly conserved and yet functionally enigmatic protein associated with a heritable form of Parkinson’s disease. It has been suggested to be a redox-dependent regulatory scaffold, binding to proteins to modulate their function. Here we present the X-ray crystal structure of the *Toxoplasma* orthologue *Toxoplasma gondii* DJ-1 (TgDJ-1) at 2.1-Å resolution and show that it directly associates with calcium-dependent protein kinase 1 (CDPK1). The TgDJ-1 structure identifies an orthologously conserved arginine dyad that acts as a phospho-gatekeeper motif to control complex formation. We determined that the binding of TgDJ-1 to CDPK1 is sensitive to oxidation and calcium, and that this interaction potentiates CDPK1 kinase activity. Finally, we show that genetic deletion of TgDJ-1 results in upregulation of CDPK1 expression and that disruption of the CDPK1/TgDJ-1 complex *in vivo* prevents normal exocytosis of parasite virulence-associated organelles called micronemes. Overall, our data suggest that TgDJ-1 functions as a noncanonical kinase-regulatory scaffold that integrates multiple intracellular signals to tune microneme exocytosis in *T. gondii*.

## IMPORTANCE

Apicomplexan parasites such as *Toxoplasma* and *Plasmodium* are obligate intracellular parasites that require the protective environment of a host cell in order to replicate and survive within a host organism. These parasites secrete effector proteins from specialized apical organelles to select and invade a chosen host cell. The secretion of these organelles is a tightly regulated process coordinated by endogenous small molecules and calcium-dependent protein kinases. We previously identified the *Toxoplasma* orthologue of the highly conserved protein DJ-1 as a regulator of microneme secretion, but the molecular basis for this was not known. We have now identified the molecular mechanism for how TgDJ-1 regulates microneme secretion. TgDJ-1 interacts with the kinase responsible for the secretion of these organelles (calcium-dependent kinase 1) and synergizes with calcium to potentiate kinase activity. This interaction is direct, phosphodependent, and necessary for the normal secretion of these important organelles.

## INTRODUCTION

Human DJ-1 (also known as PARK7) is a highly conserved, yet enigmatic protein mutated in some familial forms of Parkinson’s disease. The structure of the human DJ-1 protein confirms that it has a papain-like protease fold (1), yet there is no clear agreement on whether it functions as bona fide protease. Specifically, the homolog from the human parasite pathogen *Toxoplasma gondii* lacks the key histidine base of the catalytic triad in the papain fold, suggesting that it has functions other than proteolysis. Although there have been reports of DJ-1 functioning as a protease (2, 3), glyoxylase (4), transcriptional coactivator (5), and antioxidant (6), some of the most compelling data suggest that DJ-1 functions as a redox-dependent chaperone (7) that interprets intracellular reactive oxygen species (ROS) signals through a hyperreactive and oxidation-sensitive cysteine located in the so-called nucleophilic elbow (8). A similarly broad function was recently proposed for the *Plasmodium falciparum* orthologue (9), where it was shown to contribute to parasite virulence. It has also been proposed that, perhaps similar to the 14-3-3 proteins (10), the DJ-1 superfamily has a variety of functions mediated through interactions with client proteins or substrates (11). Although present in all domains of life (12), the molecular basis for interactions with client proteins and how it regulates the processes they modulate remain largely unknown.

Intracellular pathogens are dependent upon their chosen host cell niche for survival. Apicomplexan parasites such as *Plasmodium* spp. and *Toxoplasma gondii* utilize a complex of apical organelles consisting of dense granules, rhoptries, and micronemes, which they deploy for release (egress), attachment, and invasion of host cells, and the establishment of the parasitophorous vacuole where the parasite resides inside the host (13). The contents of these organelles include key molecules for interacting with the host cell and, correspondingly, their secretion is tightly regulated (14). Microneme secretion is perhaps the best-understood, with calcium triggering a cascade of events that result in the activation of calcium-dependent protein kinase 1 (CDPK1) and microneme secretion (15). Prior to egress, micronemes are thought to release components that enable the parasite to escape from the parasitophorous vacuole (16). Once extracellular, continued release of microneme proteins allows tachyzoites to attach to cells, to move, and to eventually invade a chosen host cell. Secretion is a calcium-driven event, which can be artificially stimulated using calcium ionophores or short-chain alcohols that trigger calcium release from internal stores (17).

In our previous work we identified a small molecule, WRR-086, that inhibited host cell invasion by *T. gondii* tachyzoites (18). WRR-086 is a peptide alpha-beta unsaturated ketone that targets the *Toxoplasma* orthologue of DJ-1. The binding of WRR-086 to a specific cysteine residue on *T. gondii* (TgDJ-1) inhibited microneme secretion and positioned TgDJ-1 in the same molecular pathway as calcium. Importantly, WRR-086 inhibition of microneme secretion could not be rescued using calcium agonists, demonstrating that, although the presence of calcium is necessary for microneme secretion, it is not sufficient. Despite the suggestion that TgDJ-1 was a novel regulator of microneme secretion, the molecular function of TgDJ-1 remained unclear.

Here, we combine structural biology, biochemistry, and cell biology to define a molecular pathway by which TgDJ-1 regulates microneme secretion. In solving the X-ray crystal structure of TgDJ-1, we identified a highly conserved arginine dyad that we confirm as being responsible for a phosphorylation-dependent interaction between TgDJ-1 and the calcium-dependent protein kinase CDPK1. Surprisingly, and despite its conservation, this arginine dyad has not been mechanistically dissected in other DJ-1 orthologues. We find that TgDJ-1 synergizes with calcium to potentiate CDPK1 kinase activity and, furthermore, that the interaction is dependent on calcium levels and the oxidation state of the environment. To confirm the functional significance of the TgDJ-1/CDPK1 complex, we engineer WRR-086-insensitive *dj-1* alleles and introduce them into wild-type (WT) parasites. These engineered alleles rescue drug-induced inhibition of microneme secretion and support a role for the complex in the regulation of this process. Together, these data suggest that through ROS, calcium, and phosphorylation-specific interactions with CDPK1, TgDJ-1 controls exocytosis of key organelles required for pathogenesis.

## RESULTS

### Structure of TgDJ-1.

**FIG 1:**
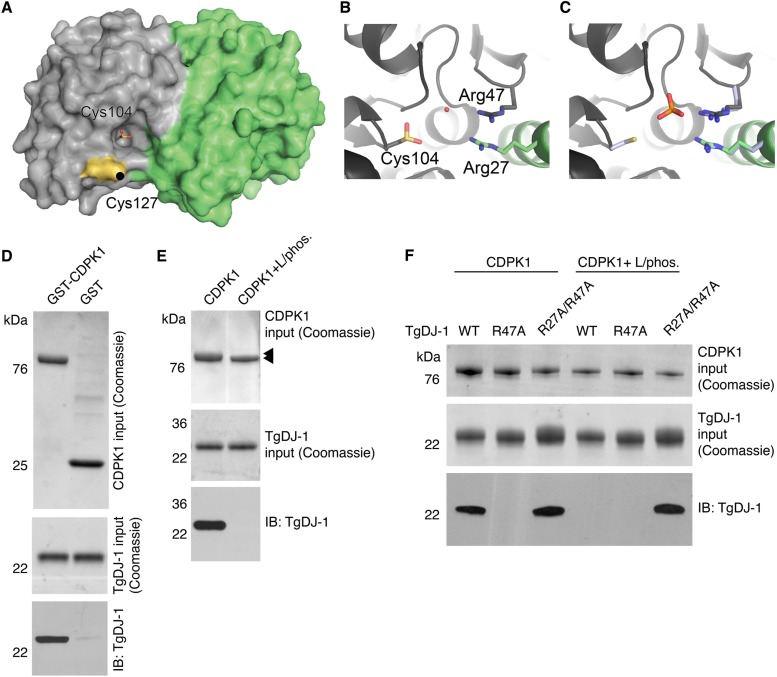
The structure of TgDJ-1 reveals a conserved arginine dyad that coordinates a direct phosphospecific interaction with CDPK1. (A) Surface representation of the TgDJ-1 structure, with the two monomers represented in gray and green. The target of WRR-086 modification, Cys127, is highlighted in yellow, and the oxidized Cys104 in the nucleophilic pocket is shown in stick representation. (B and C) The Arg27/Arg47 dyad coordinates a water molecule with the oxidized Cys104 (B), which is replaced by a PO_4_^3−^ in the structure of reduced HsDJ-1 (PDB accession no. 3BWE) (C). The structure of HsDJ-1 was overlaid with TgDJ-1, and a portion is shown, with the residues equivalent to Arg27/Arg47 shown in light blue. For clarity, TgDJ-1 Cys104 is not shown in panel C. (D) Coomassie and Western blot analysis of pulldown of TgDJ-1 by GST-CDPK1 or GST alone. (E) Coomassie and Western blot analysis of pulldown of TgDJ-1 using either GST-CDPK1 (CDPK1) or GST-CDPK1 coexpressed with lambda phosphatase (CDPK1 + L/phos.). Arrowheads indicate size shifts for the two purified GST-tagged CDPK1 species. (F) Coomassie and Western blot analysis of pulldown of wild-type TgDJ-1 (WT), TgDJ-1^R47A^ (R47A), or TgDJ-1^R27A/R47A^ (R27A/R47A) using either GST-CDPK1 (CDPK1) or GST-CDPK1 coexpressed with lambda phosphatase (CDPK1 + L/phos.). IB, immunoblot.

We previously discovered that TgDJ-1 functions within the pathway underlying the exocytosis of specialized virulence-associated organelles called micronemes (18), but the molecular mechanism by which it asserted control over this event remained unclear. To get a better sense of structural features important for TgDJ-1 function, we solved the crystal structure of TgDJ-1 to 2.1 Å (Fig. 1A and Table S4 in the supplemental material). As with other orthologues, TgDJ-1 is a constitutive homodimer. The target of WRR-086, Cys127, sits next to the nucleophilic pocket that contains Cys104 (Fig. 1A). As reported for most DJ-1 structures, the reactive Cys104 was found exclusively in the oxidized sulfinic acid state. The function of DJ-1 as a redox sensor has been linked to this highly conserved and reactive cysteine at position 106 in humans (12). To independently assess the reactivity of Cys104 in TgDJ-1, we applied a recently described method that uses cysteine sensitivity to iodoacetamide alkylation (at two concentrations, high and low) to profile the reactivity of cysteines (19). Using this approach, the more reactive a cysteine residue is, the closer its ratio score (high concentration/low concentration) is to 1. We found that TgDJ-1 Cys104 had a cysteine reactivity ratio score of 2.03 (standard deviation [SD] = 0.03), compared with the nearby reactive cysteine, cysteine 127, that is the target of WRR-086, which had a score of 3.44 (SD = 0.21). These data indicate that TgDJ-1 Cys104 is highly reactive, potentially explaining its hyperoxidized state in the crystal structure. To precisely define the reactivity of Cys104, we measured its p*K*_a_. We monitored the absorption of the thiolate anion at 240 nm as a function of pH (20, 21) and determined that it has a p*K*_a_ of 5.0 ± 0.1 (see Fig. S1 in the supplemental material). This result further confirms the high reactivity of this residue and suggests that at physiological pH, this cysteine would be highly sensitive to oxidation and could function as a ROS sensor as suggested in other systems (8).

10.1128/mBio.02189-16.1 FIG S1  Measurement of TgDJ-1 cysteine 104 p*K*_a_. The p*K*_a_ of TgDJ-1 was determined by monitoring the absorption of the thiolate anion at 240 nm as a function of pH. WT, wild-type protein; C127S, control where a nonconserved cysteine was mutated to a serine; C104S, control to demonstrate that the value being measured was being generated by C104. Download FIG S1, TIF file, 0.8 MB. Copyright © 2017 Child et al. 2017 Child et al. This content is distributed under the terms of the Creative Commons Attribution 4.0 International license.

10.1128/mBio.02189-16.6 TABLE S1  Data for the *post hoc* pairwise comparison of levels of basal microneme secretion as shown in Fig. 5B performed using Tukey’s multiple-comparison test. ns, not significant. *, *P* ≤ 0.05. **, *P* ≤ 0.01. ***, *P* ≤ 0.001. ****, *P* ≤ 0.0001. Download TABLE S1, DOCX file, 0.1 MB. Copyright © 2017 Child et al. 2017 Child et al. This content is distributed under the terms of the Creative Commons Attribution 4.0 International license.

10.1128/mBio.02189-16.7 TABLE S2  Data for the *post hoc* pairwise comparison of levels of induced microneme secretion as shown in Fig. 5D performed using Tukey’s multiple-comparison test. ns, not significant. *, *P* ≤ 0.05. **, *P* ≤ 0.01. ***, *P* ≤ 0.001. ****, *P* ≤ 0.0001. Download TABLE S2, DOCX file, 0.1 MB. Copyright © 2017 Child et al. 2017 Child et al. This content is distributed under the terms of the Creative Commons Attribution 4.0 International license.

10.1128/mBio.02189-16.8 TABLE S3  Data for the *post hoc* pairwise comparison of induced microneme secretion in the presence of titration of WRR-086 as shown in Fig. 6E, performed using Tukey’s multiple-comparison test. ns, not significant. *, *P* ≤ 0.05. **, *P* ≤ 0.01. ***, *P* ≤ 0.001. ****, *P* ≤ 0.0001. Download TABLE S3, DOCX file, 0.1 MB. Copyright © 2017 Child et al. 2017 Child et al. This content is distributed under the terms of the Creative Commons Attribution 4.0 International license.

10.1128/mBio.02189-16.9 TABLE S4  Data collection and refinement statistics. Download TABLE S4, DOCX file, 0.04 MB. Copyright © 2017 Child et al. 2017 Child et al. This content is distributed under the terms of the Creative Commons Attribution 4.0 International license.

Comparing our structure to previously deposited structures of other DJ-1 family members, we noted that the two structures of human DJ-1 in which Cys106 is reduced contain a tetragonal anion (sulfate [PDB accession no. 2OR3] [22] or phosphate [PDB accession no. 3BWE] [23]). This anion is most prominent in structures of reduced DJ-1, as it would clash with the oxidized cysteine. We noted that the ion is coordinated by a highly conserved arginine dyad present in the DJ-1 superfamily of proteins (12) (Fig. 1B and C). An interesting structural feature is that each arginine of the dyad is contributed by a different individual monomer unit of the DJ-1 dimer. Despite this high level of conservation, no function for these residues has been reported for any of the DJ-1 orthologues, including human.

### TgDJ-1 interacts with CDPK1 in a phosphodependent manner.

DJ-1 has been suggested to interact with a variety of proteins (8), leading to speculation that it functions as a scaffold or chaperone. One of the first proteins shown to directly interact with human DJ-1 was named DJ-1 binding protein (DJBP) (24), later renamed EF-hand calcium-binding domain-containing protein 6. That HsDJ-1 can bind to a calcium-responsive protein is particularly interesting as TgDJ-1 plays a role in the calcium-triggered process of microneme secretion. CDPK1 is essential for microneme secretion (15) and has an autoinhibitory C-terminal calmodulin-like domain containing four tandem EF-hand domains (25) that undergo a dramatic conformational rearrangement upon calcium binding to activate the kinase. This led us to hypothesize that TgDJ-1 could regulate microneme secretion directly through an interaction with CDPK1. To test this, we performed *in vitro* association studies using recombinant glutathione *S*-transferase (GST)-tagged CDPK1 and recombinant TgDJ-1 (rTgDJ-1). These studies confirmed that GST-CDPK1, and not GST alone, was able to precipitate rTgDJ-1, demonstrating that these two proteins can interact directly and that the complex requires no additional factors for formation (Fig. 1D).

Many kinases are regulated by autophosphorylation (26). CDPK1 contains at least three major phosphosites (serine 25, 61, and 349), identified in a recent phosphoproteomic study (27). In light of this and the insights gleaned from the crystal structure, we investigated whether phosphorylation of CDPK1 was critical for its interaction with TgDJ-1. Coexpression of CDPK1 with lambda phosphatase in *Escherichia coli* produces homogenously dephosphorylated recombinant kinase for crystallographic studies (25). The previous successful crystallization of CDPK1 coexpressed with lambda phosphatase reassured us that the protein would likely be correctly folded but lack phosphorylation modifications. Using this protocol, we expressed and purified dephosphorylated GST-tagged CDPK1. Conversion to the dephosphorylated state was confirmed by a modest SDS-PAGE shift of the GST-CDPK1 expression product (Fig. 1E). We then tested the ability of either phosphorylated or dephosphorylated CDPK1 to precipitate rTgDJ-1. Remarkably, the interaction displayed absolute dependence upon the phosphostate of the kinase, with TgDJ-1 associating only with a phosphorylated form of CDPK1 (Fig. 1E).

The TgDJ-1 crystal structure led us to consider a possible function for the highly conserved arginine dyad as a phosphate-coordinating motif located on the edge of the nucleophilic trough. As both arginine residues are expected to be required for correct coordination of the tetragonal anion, we predicted that mutation of a single arginine of the dyad would prevent correct coordination of the charged phosphate group and abrogate binding. To directly test this, we mutated the two arginine residues at positions 27 and 47 to alanine. We expressed and purified a single mutant, TgDJ-1^R47A^, and the double mutant, TgDJ-1^R27A/R47A^, and then tested whether these residues were involved in the interaction. Our attempts to generate the single TgDJ-1^R27A^ mutant were unsuccessful. As we had previously observed, the TgDJ-1 wild-type strain (TgDJ-1^WT^) interacted only with phosphorylated CDPK1 (Fig. 1F). As expected, when a single arginine of the dyad was mutated to an alanine in TgDJ-1^R47A^, CDPK1 was unable to interact regardless of its phosphorylation state (Fig. 1F). In contrast, the double mutant (TgDJ-1^R27A/R47A^) was able to bind to CDPK1 regardless of its phosphorylation state (Fig. 1F). These data are consistent with the TgDJ-1 arginine dyad functioning as a phosphorylation recognition motif or gatekeeper, facilitating recognition of phosphorylated CDPK1 by TgDJ-1.

### The association of TgDJ-1 with CDPK1 is context dependent.

**FIG 2:**
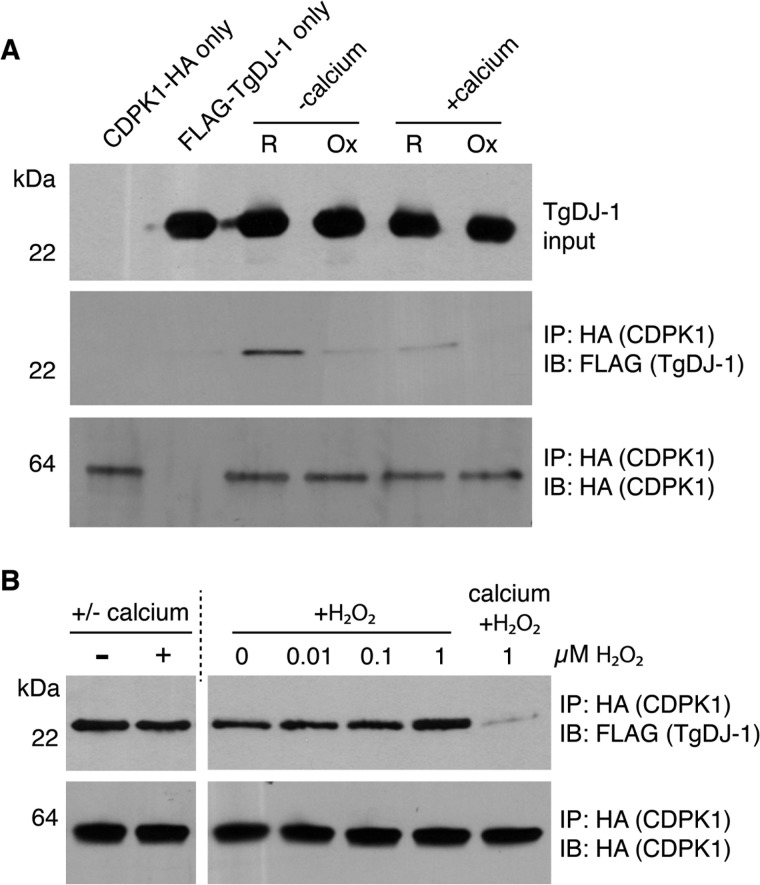
The association and stability of the TgDJ-1/CDPK1 complex are sensitive to secondary messengers. (A) Western blot analysis of the ability of HA-tagged CDPK1 to coprecipitate FLAG-tagged TgDJ-1 under low-calcium (+5 mM EGTA) (−calcium) or high-calcium (+5 mM CaCl_2_) (+calcium) conditions and reductive (R) or oxidative (Ox) conditions. IP, immunoprecipitation. IB, immunoblot. (B) Western blot analysis of the stability of isolated TgDJ-1/CDPK1 complex in the presence of 5 mM calcium (± calcium), hydrogen peroxide (+H_2_O_2_), or both.

In light of the published role for DJ-1 in sensing intracellular reactive oxygen species (28) and for CDPK1 as a calcium sensor (25), we sought to test if these naturally occurring signaling molecules could influence the interaction of the two proteins. For this purpose, we used a more authentic “cell extract” system in which we manipulated the calcium and redox conditions. We prepared soluble cell extracts from naturally egressed CDPK1-hemagglutinin (CDPK1-HA) tachyzoites and adjusted the extracts to make them either calcium rich (+calcium) or calcium depleted (+EGTA), as well as reducing (+dithiothreitol [+DTT]) or oxidizing (+H_2_O_2_). We used H_2_O_2_ as the oxidizing agent as it is broadly regarded as the most relevant candidate for the transduction of ROS signals (29). After addition of recombinant TgDJ-1 (rTgDJ-1), we immunoprecipitated (IP) CDPK1-HA and assessed the ability of the kinase to coprecipitate rTgDJ-1. Interestingly, the interaction between rTgDJ-1 and CDPK1-HA was both calcium and H_2_O_2_ dependent (Fig. 2A), with rTgDJ-1 predominantly interacting with CDPK1 in reducing conditions and in the absence of calcium.

We next sought to determine if specific signals could trigger dissociation of the TgDJ-1/CDPK1 complex. We isolated the complex from soluble cell extracts using conditions that favored its assembly (reducing and calcium depleted) and then incubated with calcium or H_2_O_2_. Interestingly, the addition of either calcium or H_2_O_2_ alone was not sufficient to trigger dissociation of the complex (Fig. 2B). However, in the presence of both calcium and ROS, the complex dissociated (Fig. 2B). These data suggest that the TgDJ-1/CDPK1 complex could function as a sensor for these two secondary messengers.

### TgDJ-1 potentiates CDPK1 activity.

**FIG 3:**
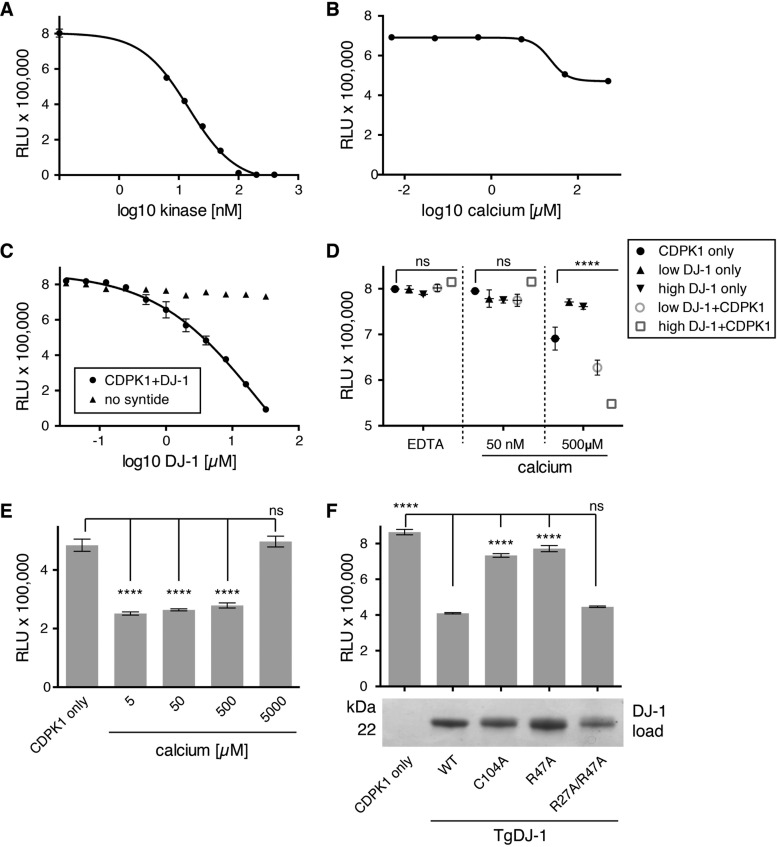
TgDJ-1 potentiates CDPK1 kinase activity, with the effect dependent upon, and synergistic with, calcium. (A) Kinase activity assay to optimize CDPK1 concentration. Scatter plot of data points from a kinase assay optimizing the concentration of CDPK1 kinase required to generate robust signal for 10 µM syntide and 10 µM ATP. (B) Kinase activity assay to optimize calcium concentration for CDPK1 activation. Scatter plot of data points from a kinase assay testing the concentration of calcium required to fully activate CDPK1. (C) Kinase activity assay testing the effect of TgDJ-1 upon CDPK1 activity. Scatter plot of data points from a kinase assay where TgDJ-1 was titrated against CDPK1, either in the presence (CDPK1+DJ-1) or absence of the peptide substrate (no syntide). (D) Kinase assay testing the effect of calcium on pre-complexed CDPK1+DJ-1. Scatter plot of kinase assay data points where CDPK1 alone (CDPK1 only), TgDJ-1 alone (DJ-1 only), or TgDJ-1+CDPK1 (DJ-1+CDPK1) was incubated with EDTA, 50 nM, or 500 µM calcium. Two concentrations of TgDJ-1 were used: 0.26 µM (low DJ-1) or 2.6 µM (high DJ-1). Significance was calculated for a two-way ANOVA statistical analysis (with Dunnett’s multiple comparison test) comparing each group of means (EDTA, 50 nM or 500 µM calcium) with the CDPK1 only control within the group. ns, not significant; ****, *P* ≤ 0.0001. (E) Kinase assay testing the effect of incubating CDPK1 with TgDJ-1 in the presence of a titration of calcium. Histogram of kinase assay data points for CDPK1 only, or TgDJ-1+CDPK1 pre-incubated with 5, 50, 500, or 5,000 µM calcium. Data presented are the means of three replicates, with error bars indicating ± SD. Significance was calculated for an ordinary one-way ANOVA statistical analysis (with Dunnett’s multiple comparison test) comparing each mean with the CDPK1 only control. ns, not significant; ****, *P* ≤ 0.0001. (F) Kinase assay testing the capacity of different TgDJ-1 mutants to potentiate CDPK1 activity. Histogram of kinase assay data points for CDPK1 alone (CDPK1 only), or CDPK1 incubated with 10 µM wild-type, C104A, R47A, or R27A/R47A mutant TgDJ-1 protein. Data presented are the means of three replicates, with error bars indicating ± SD. Significance was calculated for an ordinary one-way ANOVA statistical analysis (with Dunnett’s multiple comparison test) comparing each mean with wild-type TgDJ-1 (WT). ns, not significant; ****, *P* ≤ 0.0001. The panel beneath the histogram is a Coomassie stained gel indicating equal input of the different TgDJ-1 mutant proteins in the kinase assay reactions. For panels A, B, C, and D, data presented are the means of three replicates, with error bars indicating ± SD. RLU, relative light units.

To determine if DJ-1 binding had any direct effect on CDPK1 function, we performed an *in vitro* kinase assay, measuring residual ATP levels present in a given reaction following phosphorylation of a peptide substrate as an endpoint read (low signal = high kinase activity). We optimized the concentration of CDPK1 based on its 50% effective concentration (EC_50_) of ~15 nM, hypothesizing that this concentration of kinase would provide sufficient dynamic range to distinguish between inhibition and activation in subsequent experiments (Fig. 3A). CDPK1 has been structurally confirmed to exist in two distinct conformers (25): apo (calcium-free, inactive) and calcium-bound (active). Intracellular concentrations of calcium in tachyzoites have been estimated to be between 50 and 100 nM (30); however, we observed kinase activity at relatively high concentrations of between 5 and 50 µM calcium (Fig. 3B). On the basis of these data, we induced binding of TgDJ-1 under low (50 nM)-calcium conditions, followed by the addition of ATP and peptide substrate and a high calcium concentration (500 µM) to stimulate full activation of the kinase. To test if TgDJ-1 affected the activity of the kinase, we titrated wild-type TgDJ-1 against CDPK1 and discovered that TgDJ-1 increased the activity of CDPK1 in a dose-dependent manner (Fig. 3C). We did not observe this effect in the absence of the peptide substrate, indicating that the effect was most likely due to increased phosphorylation of the peptide rather than to increased CDPK1 autophosphorylation or phosphorylation of TgDJ-1.

We then tested whether the presence of TgDJ-1 was sufficient to activate CDPK1, whether activation still required calcium, and if any effect observed was additive or synergistic. We performed an assay where different concentrations of TgDJ-1 (low, 0.26 µM; high, 2.6 µM) were added to CDPK1, followed by the addition of the peptide substrate, ATP, and 500 µM EDTA or a low (50 nM) or high (500 µM) concentration of calcium. Neither the EDTA-treated samples nor the low-calcium-treated samples exhibited any appreciable activity, and this was unaffected by the presence of TgDJ-1 (Fig. 3D, EDTA and 50 nM sections of scatter plots). We once again observed that high concentrations of calcium activated the kinase and that TgDJ-1 stimulated this activity in a dose-dependent manner (Fig. 3D; 500 µM section of scatter plot). Thus, the calcium and TgDJ-1 effects on CDPK1 activity appear to be synergistic in nature. Assays performed with TgDJ-1 in the absence of CDPK1 produced no signal, confirming that the observed signals for the TgDJ-1-plus-CDPK1 (TgDJ-1+CDPK1) samples were not due to contaminants in the TgDJ-1 recombinant protein preparations (Fig. 3D).

We hypothesized that the activation observed with different concentrations of calcium was a reflection of CDPK1 transitioning from the apo state to the fully active conformer (Fig. 3B) (25). To investigate how these different calcium-bound CDPK1 conformers responded to TgDJ-1 potentiation, TgDJ-1 was allowed to associate with CDPK1 in the presence of a titration of calcium. We observed that in high concentrations of calcium, the potentiation effect of TgDJ-1 was blocked, with kinase activity returning to the level seen with kinase alone (Fig. 3E).

Combining our insights from the crystal structure and the precipitation data, we hypothesized that if the association of TgDJ-1 with CDPK1 were necessary to potentiate kinase activity, TgDJ-1 mutations predicted or directly shown to affect the interaction could alter its ability to influence kinase activity. We assayed a panel of TgDJ-1 mutants for their ability to potentiate CDPK1 activity. These included the arginine dyad mutants (R47A, R27A/R47A) and an additional mutant where the reactive cysteine C104 was mutated to an alanine (C104A) that we predicted could participate in the interaction on the basis of the crystal structure. Consistent with our precipitation data, the R47A mutant that failed to associate with CDPK1 (Fig. 1F) was defective in its ability to potentiate CDPK1 activity (Fig. 3F). The double-arginine mutant (R27A/R47A) that constitutively associated with CDPK1 irrespective of its phosphorylation state (Fig. 1F) potentiated kinase activity to a degree similar to that seen with wild-type TgDJ-1 (Fig. 3F). Interestingly, the C104A mutant displayed a similar loss in its ability to potentiate kinase activity, indicating that this critical and highly conserved residue may also contribute to the interaction.

### TgDJ-1 regulates microneme secretion.

**FIG 4:**
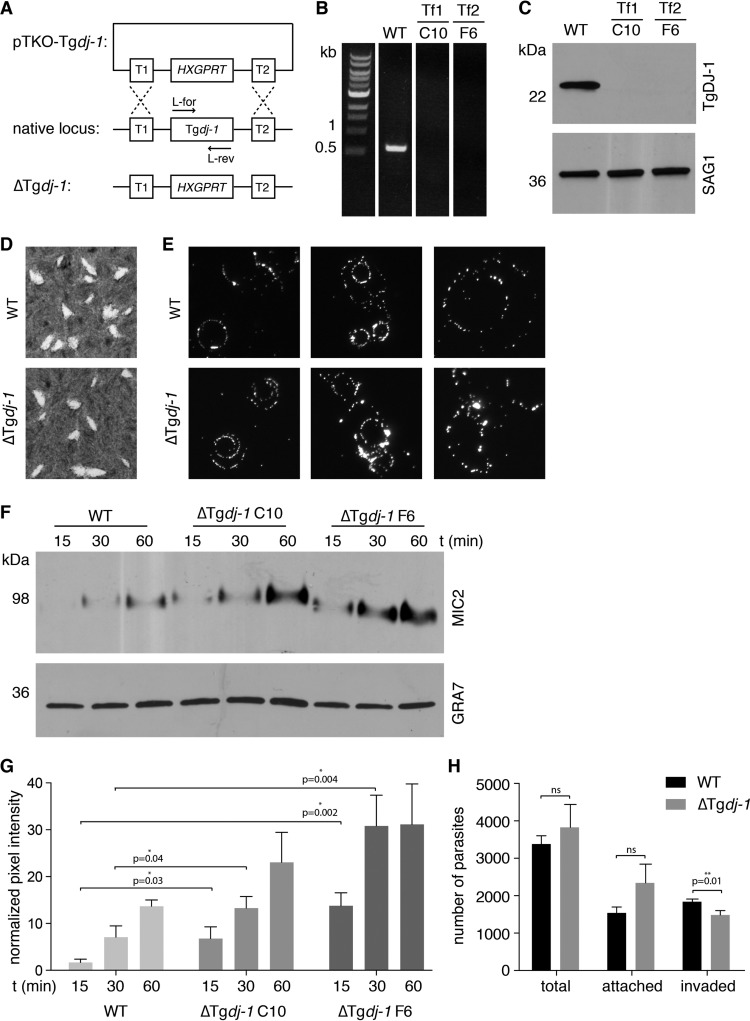
Phenotypic characterization of ΔTg*dj-1* parasites. (A) Strategy to replace the native *dj-1* locus with the drug-selectable *HXGPRT* cassette and generate the ΔTg*dj-1* parasite line. (B) PCR confirmation of loss of the *dj-1* locus in two individual clones (C10 and F6) isolated from two independent transfections (Tf1 and Tf2). (C) Western blot confirmation of the loss of TgDJ-1 protein expression in the same ΔTgDJ-1 parasite clones. SAG1, loading control. (D) Plaque assays comparing growth of wild-type (WT) and TgDJ-1 knockout (ΔTgDJ-1) parasites. (E) Gliding motility assays comparing the 2D motility of wild-type (WT) and TgDJ-1 knockout (ΔTg*dj-1*) parasites. (F) Microneme secretion assays comparing the levels of basal secretion of MIC2 over time for the wild-type (WT) strain and two individual ΔTgDJ-1 clones (C10 and F6) isolated from two independent transfections. GRA7, loading control. (G) Quantification of the microneme secretion assays. Asterisk indicates the level of significance as determined by Student’s *t* test; data represent means ± SD for results from *n* = 3 experiments. (H) Attachment/invasion assay comparing wild-type (WT) and TgDJ-1 knockout (ΔTg*dj-1*) parasites. The histogram presents the quantification of raw parasite numbers for attachment/invasion assays for the wild-type (WT) strain versus ΔTg*dj-1* clone F6. Asterisk indicates the level of significance as determined by Student’s *t* test; data represent means ± SD for results from *n* = 3 experiments.

Given the structural and functional data suggesting a potential biological role for the DJ-1/CDPK1 complex, we moved to an intact cell system to validate the relevance of the complex. We began by deleting the native *dj-1* (TGGT1_214290) locus using double homologous recombination in the RHΔ*ku80* parasite line to replace the gene with the *HXGPRT* drug selection cassette (Fig. 4A). We confirmed successful deletion of the *dj-1* locus by diagnostic PCR analysis (Fig. 4B). To better assess the knockout parasites (the ΔTg*dj-1* mutants), we raised a murine polyclonal antibody against *E. coli-*expressed recombinant TgDJ-1 (18). When used to probe Western blots of whole tachyzoite lysates, the antibody recognized a single species of ~22 kDa (Fig. S2A), consistent with its predicted size of 19.5 kDa, and biochemical fractionation showed that TgDJ-1 partitioned exclusively to the cytosolic compartment (Fig. S2B). This result was supported by its localization in immunofluorescence assays (Fig. S2C). We isolated clonal populations of knockout parasites by limiting dilution, and Western blot analysis confirmed the absence of the TgDJ-1 protein (Fig. 4C). Plaque assays indicated that ΔTg*dj-1* parasites grew normally in terms of both plaque number and size (Fig. 4D), suggesting that, in this genetic background and under the conditions used for cell culture, TgDJ-1 is nonessential or can be readily compensated for. Likewise, the motility of ΔTg*dj-1* parasites in two dimensions (2D) was indistinguishable from the motility of the wild type (Fig. 4E).

10.1128/mBio.02189-16.2 FIG S2  Murine polyclonal antibody specific for TgDJ-1 defines its biochemical properties and subcellular localization. (A) Western blot analysis of *T. gondii* tachyzoite lysates, probed with murine serum isolated pre- and postimmunization with recombinant TgDJ-1 (lanes labeled preimmune and final bleed, respectively). (B) Biochemical fractionation of soluble and insoluble fractions of *T. gondii* tachyzoites, followed by Western blotting of those fractions. An osmotic fractionation protocol was used to separate the insoluble membrane/cytoskeleton fraction from the soluble fraction. (C) Indirect immunofluorescence assays examining the localization of TgDJ-1 in intra- and extracellular parasites. GAP45, gliding-associated protein 45, used as a marker for the IMC/plasma membrane; SAG1, surface antigen 1, used as a marker for the plasma membrane. DAPI, nuclear stain. Scale bar, 5 µM. Download FIG S2, TIF file, 0.6 MB. Copyright © 2017 Child et al. 2017 Child et al. This content is distributed under the terms of the Creative Commons Attribution 4.0 International license.

**FIG 5:**
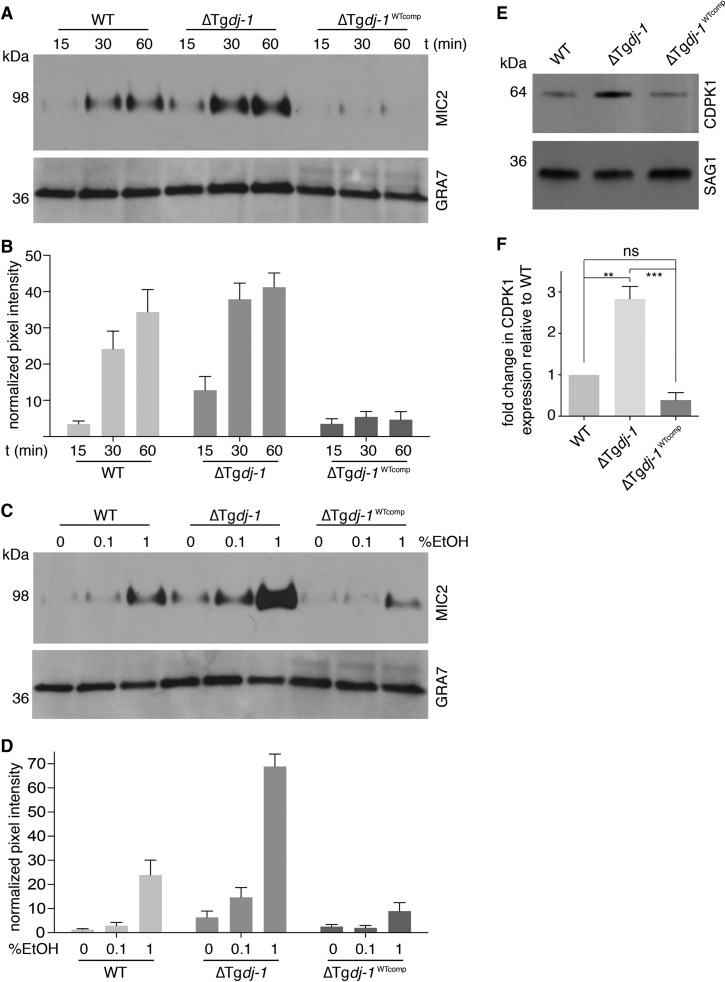
TgDJ-1 regulates microneme secretion. (A) Western blots of microneme secretion assays comparing levels of basal secretion of MIC2 over time for wild-type (WT), ΔTg*dj-1* clone F6, and ΔTg*dj-1* parasites complemented with wild-type Tg*dj-1* (ΔTg*dj-1*^WTcomp^). GRA7, loading control. (B) Quantification of basal microneme secretion assays; data represent means ± SD for results from *n* = 3 experiments. Significance was calculated for a two-way statistical ANOVA for comparison of the different strains (WT, ΔTg*dj-1*, and ΔTg*dj-1*^WTcomp^) (****, *P* ≤ 0.0001) and the different time points (15, 30, and 60 min) (****, *P* ≤ 0.0001). *Post hoc* pairwise comparisons of the different strains at each time point were performed using Tukey’s multiple-comparison test and the data presented in Table S1. (C) Western blots of microneme secretion assays comparing ethanol-induced secretion of MIC2 with different concentrations of ethanol for the wild-type strain (WT), the TgDJ-1 knockout strain (ΔTgDJ-1), and the TgDJ-1 knockout strain complemented with wild-type TgDJ-1 (ΔTgDJ-1^WTcomp^). GRA7, loading control. (D) Quantification of induced microneme secretion assays; data represent means ± SD for results from *n* = 3 experiments. Significance was calculated for a two-way statistical ANOVA for comparison of the different strains (WT, ΔTg*dj-1*, and ΔTg*dj-1*^WTcomp^) (****, *P* ≤ 0.0001) and the different proportions of EtOH (0%, 0.1%, and 1%) (****, *P* ≤ 0.0001). *Post hoc* pairwise comparisons of the different strains at each ethanol concentration were performed using Tukey’s multiple-comparison test and the data presented in Table S2. (E) Western blot comparing expression levels of CDPK1 in wild-type (WT), ΔTg*dj-1*, and ΔTg*dj-1*^WTcomp^ parasites. SAG1, loading control. (F) Quantification of CDPK1 expression levels. Data represent means ± SD for results from *n* = 3 experiments. Significance was calculated for an ordinary one-way statistical ANOVA to assess the difference between the means (***, *P* ≤ 0.001). Results of a *post hoc* Tukey’s multiple-comparison test for pairwise comparisons of wild-type (WT), Tg*dj-1*, and ΔTg*dj-1*^WTcomp^ parasites are presented. ns, not significant. **, *P* ≤ 0.01. ***, *P* ≤ 0.001.

The original identification of TgDJ-1 as the target of the small molecule WRR-086 suggested that it was involved in the regulation of microneme secretion (18). With this in mind, we investigated if microneme secretion was altered in ΔTg*dj-1* parasites. Microneme secretion can be monitored over time by detection of microneme protein 2 (MIC2) in culture supernatant following its release by organelle exocytosis. Basal microneme secretion in two knockout clones selected from two independent transfections (clones C10 and F6) was increased compared to that seen with the RHΔ*ku80* parent (WT) line (Fig. 4F and G and 5A and B). As both of the ΔTg*dj-1* clones exhibited the same phenotype, we continued our studies focusing on clone F6. Consistent with atypical microneme secretion, ΔTg*dj-1* parasites were moderately less proficient at invading host cells during a time-restricted attachment/invasion assay (Fig. 4H). As microneme secretion provided the clearest readout for the phenotype resulting from the loss of TgDJ-1, we focused on this assay for the remainder of the study. To determine if the absence of TgDJ-1 was the only factor responsible for the microneme secretion defect, we performed a genetic rescue through complementation performed with an N-terminally FLAG-tagged TgDJ-1 protein expressed under the control of the *GRA1* promoter and targeted to the *UPRT* locus of ΔTg*dj-1* parasites (ΔTg*dj-1*^WTcomp^) (Fig. S3A). Expression of FLAG-tagged DJ-1 was confirmed by Western blotting (Fig. S3B), and a single clone (D3) selected for further characterization. Surprisingly, ΔTg*dj-1*^WTcomp^ parasites had reduced levels of basal microneme secretion compared to both wild-type and knockout parasites (Fig. 5A and B). As there are two distinct modes of microneme secretion, we next tested whether induced secretion was similarly affected by complementing the transgene. Ethanol-stimulated microneme secretion in wild-type parasites was induced to a higher level in ΔTg*dj-1* parasites than in the parental strain (Fig. 5C and D). Furthermore, the rescue of TgDJ-1 expression in the complemented ΔTg*dj-1*^WTcomp^ cell line decreased the response of secretion to ethanol stimulation, reducing it to levels below those seen with WT parasites (Fig. 5C and D). In an attempt to better understand these results, we examined the expression levels of TgDJ-1 and found that it was dramatically overexpressed in the ΔTg*dj-1*^WTcomp^ parasites compared to native TgDJ-1 expression levels (Fig. S3C). This was likely due to the use of the *GRA1* promoter to drive its expression. Despite the observed differences in microneme secretion, ΔTg*dj-1*^WTcomp^ parasites grew normally in terms of plaque number and size (Fig. S3D). Combined, these data confirm that TgDJ-1 plays a regulatory role in both basal and induced microneme secretion.

10.1128/mBio.02189-16.3 FIG S3  Complementation of ΔTg*dj-1* parasites with FLAG-Tg*dj-1*. (A) Scheme to target and replace the *uprt* locus with a FLAG-tagged TgDJ-1 expression cassette in the background of the ΔTg*dj-1* parasites to generate the ΔTg*dj-1*^WTcomp^ parasite line. (B) Western blot analysis of **Δ**Tg*dj-1*^WTcomp^ clones isolated by limiting dilution. ΔTg*dj-1*, negative control. (C) Western blot analysis comparing the expression levels of TgDJ-1 in wild-type (WT), ΔTg*dj-1*, and ΔTg*dj-1*^WTcomp^ parasites. CDPK1, loading control. (D) Plaque assays comparing growth levels of wild-type (WT) and ΔTg*dj-1* parasites complemented with wild-type Tg*dj-1* (ΔTg*dj-1*^WTcomp^). Download FIG S3, TIF file, 4 MB. Copyright © 2017 Child et al. 2017 Child et al. This content is distributed under the terms of the Creative Commons Attribution 4.0 International license.

To confirm that the TgDJ-1/CDPK1 interaction occurred in cells, we performed coimmunoprecipitations (co-IPs) using parasite strains expressing epitope-tagged versions of TgDJ-1 (FLAG-TgDJ-1 in ΔTg*dj-1*^WTcomp^ parasites) to pull down endogenous CDPK1 or parasite strains expressing hemagglutinin-tagged CDPK1 (CDPK1-HA) (31) to pull down endogenous TgDJ-1. Though suggestive of an interaction, the results from these coimmunoprecipitations were inconsistent, leading us to speculate that the interaction may be of low affinity or that the context dependence of the interaction was too sensitive or transient to capture in these coimmunoprecipitations.

On the basis of the known role for CDPK1 as an essential regulator of microneme secretion (15), our data showing that TgDJ-1 interacts with CDPK1 (Fig. 1D and E and 2), and the observed microneme secretion phenotype in the ΔTg*dj-1* and ΔTg*dj-1*^WTcomp^ parasites (Fig. 4F and G and 5A and B and C and D), we examined the levels of CDPK1 expression in these different parasite lines. Intriguingly, CDPK1 protein levels showed a striking inverse relationship to the amount of TgDJ-1 present in the cell; in the absence of TgDJ-1, CDPK1 was more abundant at the protein level, with the amount of CDPK1 being ~3 times greater in ΔTg*dj-1* parasites than in the wild type (Fig. 5E and F). To determine if this was a general mechanism by which parasites coped with the loss of TgDJ-1, we assessed the level of CDPK1 expression in a second ΔTg*dj-1* clone isolated from an independent transfection and found that it was similarly increased (Fig. S4). These data suggested that compensating for the loss of TgDJ-1 by increasing the amount of CDPK1 present in the cell is a potential mechanism of adjustment within the pathway. In agreement with this, CDPK1 levels in ΔTg*dj-1*^WTcomp^ parasites were lower than in the knockout strain (Fig. 5E and F).

10.1128/mBio.02189-16.4 FIG S4  Upregulation of CDPK1 in response to the loss of TgDJ-1 is a general compensatory mechanism. Western blot analysis of the expression of CDPK1 in wild-type parasites (WT) in comparison to two ΔTgDJ-1 clones selected from individual transfections (F6 and C10). SAG1, loading control. Download FIG S4, TIF file, 0.7 MB. Copyright © 2017 Child et al. 2017 Child et al. This content is distributed under the terms of the Creative Commons Attribution 4.0 International license.

### Drug-insensitive *dj-1* alleles enable dissection of mutant function in cells.

**FIG 6:**
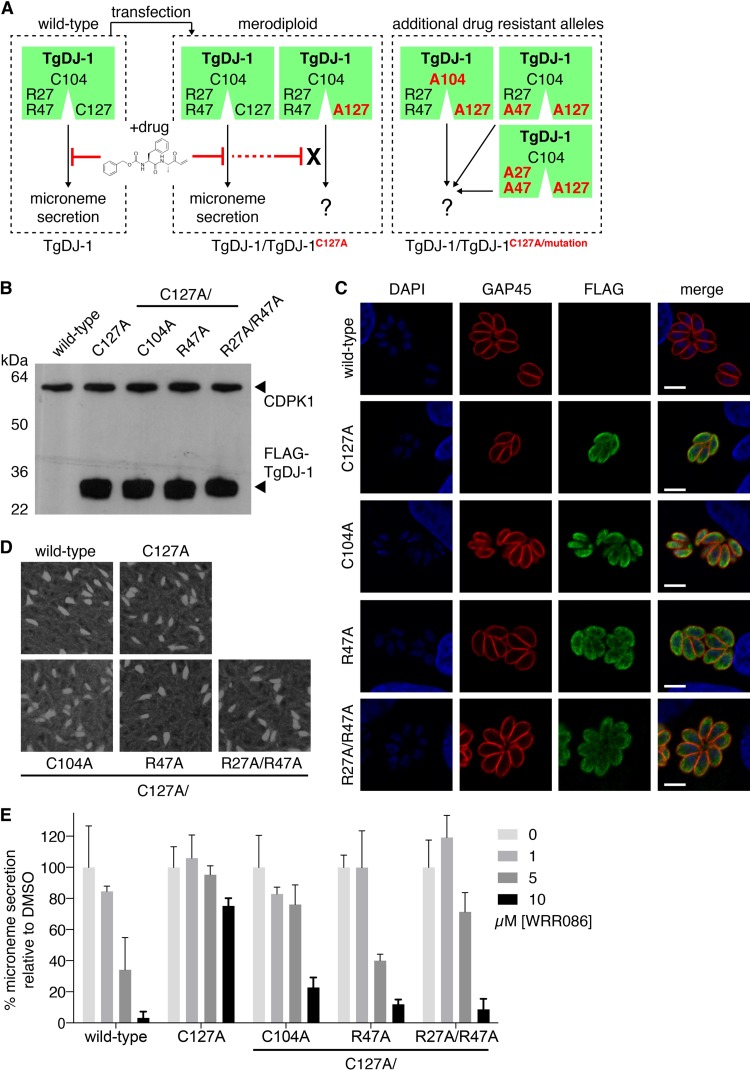
Engineered drug-insensitive TgDJ-1 alleles confirm the functional contribution of the TgDJ-1/CDPK1 complex to microneme secretion. (A) Schematic of the strategy to engineer and introduce drug-insensitive alleles to generate merodiploid parasite strains. (B) Western blot analysis using both anti-CDPK1 and anti-FLAG antibodies for expression of CDPK1 and FLAG-tagged TgDJ-1 in isogenic parasite strains expressing either TgDJ-1C127A (C127A), TgDJ-1C127A/C104A (C104A), TgDJ-1C127A/R47A (R47A), or TgDJ-1C127A/R27A/R47A (R27A/R47A). This nomenclature for the merodiploid parasites is used throughout. (C) Indirect immunofluorescence assays examining the localization of intracellular TgDJ-1 merodiploid parasites. Gliding-associated protein 45 (GAP45) is used as a marker for the inner-membrane complex/plasma membrane; DAPI, nuclear stain. Scale bar = 5 µM. (D) Plaque assays comparing growth of wild-type and TgDJ-1 merodiploid parasites. (E) Quantification of Western blot analysis of induced microneme secretion assays performed in the presence of a titration of WRR-086. Data represent means ± SD for *n* = 3 experiments. Significance was calculated for a two-way ANOVA statistical analysis comparing wild-type and all merodiploid strains. Significance for comparison of the different strain group means = **** (*P* ≤ 0.0001); and the different WRR-086 concentrations (0, 1, 5, 10 µM) = **** (*P* ≤ 0.0001). Post-hoc pairwise comparison of the different strains for each WRR-086 concentration was performed using Tukey’s multiple comparison test and the data presented in Table S3.

We sought to test if mutations that affected the DJ-1/CDPK1 complex and the ability of TgDJ-1 to potentiate CDPK1 activity also influenced its ability to regulate microneme exocytosis. Initial attempts to introduce mutant *dj-1* alleles into parasites proved unsuccessful, suggesting that the arginine dyad mutations were not tolerated when expressed in ΔTg*dj-1* parasites, eliminating the possibility of examining the function of these mutations by direct gene replacement strategies. To overcome this technical problem, we attempted to generate *dj-1* alleles N-terminally tagged with an FKBP-derived destabilization domain (32). However, the incorporation of this domain resulted in a fusion protein that was not stably expressed. In our previous work, we demonstrated that the small molecule WRR-086 targets a nonconserved cysteine at position 127 (Cys127) (18). Mutation of this cysteine in the native *dj-1* locus protected parasites from the effect of the drug. This led us to consider an alternative strategy using the C127A mutation to engineer drug-insensitive *dj-1* alleles that could be expressed in the background of wild-type TgDJ-1, making the parasites *merodiploid* for the gene. The wild-type allele could then be selectively targeted with WRR-086, allowing any phenotype associated with other mutations carried on the C127A background to be revealed through complementation (Fig. 6A). We anticipated that these mutant proteins would be phenotypically dominant in the presence of drug, allowing us to test the functional contribution of the mutations found to affect the ability of TgDJ-1 to potentiate CDPK1 activity *in vitro* with kinase function in cells. We expected that any mutation that affected the nature of the interaction would be unable to functionally complement the loss of the wild-type protein.

We transfected tachyzoites with constructs to overexpress a FLAG-tagged TgDJ-1^C127A^ mutant allele in the background of the endogenous TgDJ-1 through use of the *GRA1* promoter to drive the transgene. Using this approach, we generated the TgDJ-1/TgDJ-1^C127A^ merodiploid line (Fig. 6A), as well as lines that expressed the C104A, R47A, and R27A/R47A mutations maintained in the background of the drug-insensitive C127A allele, referred to as TgDJ-1/TgDJ-1^C127A/C104A^, TgDJ-1/TgDJ-1^C127A/R47A^, and TgDJ-1/TgDJ-1^C127A/R27A/R47A^, respectively. We isolated isogenic populations of parasites expressing FLAG-tagged TgDJ-1 mutant alleles and confirmed equivalent relative expression levels of the mutant proteins by Western blotting (Fig. 6B). Unlike the results seen with the ΔTgDJ-1 parasites, we found that the levels of CDPK1 were unaffected by the introduction of these mutant proteins (Fig. 6B). Indirect immunofluorescence assay (IFA) results confirmed a diffuse cytosolic distribution for the overexpressed mutant proteins that matched that observed for native TgDJ-1 (Fig. 6C and S2C). All merodiploid TgDJ-1 parasite lines grew normally, generating plaques that were equal in number and size to wild-type plaques (Fig. 6D). Together, these data confirmed that the mutant proteins were expressed and localized appropriately to potentially complement the native gene product. We next tested the ability of the mutant alleles to rescue inhibition of microneme secretion by WRR-086, and, importantly, microneme secretion was similar to wild-type secretion levels in the absence of drug for all merodiploid lines (Fig. S5). As anticipated, TgDJ-1/TgDJ-1^C127A^ parasites were resistant to the effect of WRR-086, exhibiting relatively normal levels of microneme secretion in the presence of the drug (Fig. 6E). It should be noted that, although TgDJ-1/TgDJ-1^C127A^ parasites were resistant to WRR-086, the level of rescue achieved was not complete, suggesting that the overexpression of the C127A allele could not fully complement the loss of function of the native protein. Parasites expressing the TgDJ-1^C127A/C104A^ allele were partially resistant to the effects of WRR-086, but the level of phenotypic rescue achieved was lower than that seen with the TgDJ-1^C127A^ allele (Fig. 6E), supporting the kinase assay data indicating a likely critical role for this residue in the potentiation of CDPK1 activity and TgDJ-1 function in microneme secretion. As expected, and in accordance with our *in vitro* biochemical data showing that the mutation of one arginine of the dyad prevented association of TgDJ-1 with the kinase, the TgDJ-1^C127A/R47A^ allele was incapable of rescuing microneme secretion in the presence of drug (Fig. 6E). Intriguingly, TgDJ-1/TgDJ-1^C127A/R27A/R47A^ parasites displayed drug sensitivity that was similar to that seen with parasites expressing the single arginine mutant (TgDJ-1/TgDJ-1^C127A/R47A^; Fig. 6E). These data confirm that mutations that disrupt or change the features of the TgDJ-1/CDPK1 complex also influence the ability of the protein to regulate microneme exocytosis *in vivo*.

10.1128/mBio.02189-16.5 FIG S5  TgDJ-1 mutant allele expression does not affect microneme secretion in the absence of WRR-086. Quantification of induced microneme secretion assays for the wild type and the different merodiploid lines. Statistical analyses of variation of means were performed by multiple comparison ANOVA; data represent means ± SD for results from *n* = 3 experiments. No merodiploid line was calculated to be significantly different from the wild type. Download FIG S5, TIF file, 0.9 MB. Copyright © 2017 Child et al. 2017 Child et al. This content is distributed under the terms of the Creative Commons Attribution 4.0 International license.

## DISCUSSION

**FIG 7:**
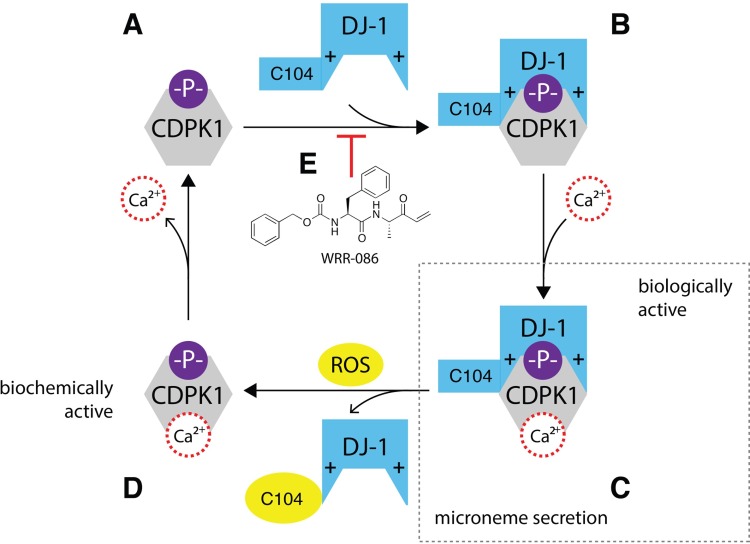
Model molecular pathway for the regulation of microneme secretion by TgDJ-1 and CDPK1. (A) CDPK1 is thought to autophosphorylate as part of its mechanism of activation. The negatively charged phosphate is indicated (-P-). (B) Under reducing conditions, TgDJ-1 associates with the phosphorylated, calcium-depleted conformer of CDPK1 to produce state B, with the negative charge of the phosphate recognized and coordinated by the conserved arginine dyad on TgDJ-1. These positively charged arginine residues are indicated (+). (C) The addition of calcium produces the final biologically active TgDJ-1/CDPK1 complex, as illustrated, with TgDJ-1 synergizing with calcium to potentiate CDPK1 activity. This potentiated complex is required to maintain normal microneme secretion. (D) The combination of a calcium and ROS signal (possibly H_2_O_2_) triggers dissociation of the complex, returning CDPK1 to the unbound, lower-activity state, allowing the kinase to recycle to reassociate with a pool of reduced TgDJ-1 protein. (E) The small-molecule inhibitor WRR-086 binds to TgDJ-1 and potentially prevents association with CDPK1. This prevents of the formation of the TgDJ-1/kinase complex shown in panel B, with the net result being that the CDPK1 cannot achieve the TgDJ-1-potentiated activity state, leading to a block in microneme secretion as previously reported (18).

The specific cellular functions of DJ-1 have remained unclear despite its association with Parkinson’s disease and cancer (8). We initiated this study on the basis of our previous finding that the *T. gondii* orthologue of DJ-1 was somehow functionally involved in the regulation of exocytosis of invasion-associated organelles called micronemes (18). Here we present a speculative molecular explanation for how TgDJ-1 can control this critical cellular event, through direct interaction with the calcium-dependent kinase CDPK1 (Fig. 7). The basis for this pathway is an association between TgDJ-1 and CDPK that is dependent on both redox and environmental calcium levels, as well on phosphorylation of the kinase. Thus, TgDJ-1 regulates the CDPK1-mediated signaling necessary for the coordination of microneme secretion.

Our findings raise the following question: how is it that the DJ-1/CDPK1 complex is stable under conditions that preclude its formation? Mechanistically, we hypothesize that TgDJ-1 locks CDPK1 in an unsaturated calcium conformer and that reactive oxygen species can promote a relaxation of the interaction by lowering the dissociation constant (*K*_*d*_) of the interaction. In this relaxed state, CDPK1 conformationally responds to a calcium signal and undergoes a further structural rearrangement with respect to the calcium-saturated conformer (25). The calcium-mediated effect is therefore robust and switch-like, with calcium triggering a rearrangement in the conformation of CDPK1 to a form that is no longer recognized by TgDJ-1, i.e., that is not competent to associate with TgDJ-1 at all. With this in mind, our data would indicate that the calcium signal is likely to be dominant. Though our data suggest that the oxidation state of TgDJ-1 could modulate complex stability, the molecular basis for this is still unclear, and the specific contribution of the reactive C104 to the association with CDPK1 is being investigated in more detail. Considering the merodiploid data for the C104A mutant, the critical comparison is the C127A mutant with the C127A/C104A mutant. These data suggest that although this mutant is still able to complement the wild-type protein function in the presence of low concentrations of drug, at the higher concentrations of WRR-086 (those at which we anticipate that the bulk of the interaction is forced to occur via the C127A allele product), the C104A mutation cannot functionally complement the native gene product, as expected on the basis of the data presented in Fig. 3F. Although TgDJ-1 and calcium seem to function synergistically to potentiate CDPK1 activity (Fig. 3D), the order of their binding to the kinase appears to be critical. Driving CDPK1 toward a calcium-saturated conformation prevents TgDJ-1 association and subsequent potentiation of kinase activity. However, it should be noted that the ability of high calcium concentrations to abrogate the activation effect of TgDJ-1 could be the result of ionic shielding of the electrostatic interactions between the arginine dyad and the phosphorylation site on CDPK1. These data are consistent with the coprecipitation data indicating that the complex could not form under calcium-rich conditions (Fig. 2A). We hypothesize that TgDJ-1 associates with CDPK1 under low-calcium conditions but that formation of this complex is not sufficient to activate the kinase. Calcium is still required and then functions synergistically with TgDJ-1 to potentiate CDPK1 activity.

The fact that coincidental detection of a calcium and ROS signal is necessary for efficient dissociation of the complex is a particularly interesting feature of the interaction. Given that formation of the complex seems to be required for maintaining normal microneme secretion, dissociation of the complex triggered by calcium and H_2_O_2_ could be a negative-feedback mechanism. Coincidental detection of these two signals would result in the complex dissociating, and CDPK1 could then be recycled back to reassociate with a reduced pool of TgDJ-1. This could produce an oscillation of CDPK1 activity. Intriguingly, parasite motility (a process driven by microneme components following exocytosis) appears to be a pulsed, oscillatory process, with the motility of extracellular parasites fluctuating in waves (33).

The high concentration of TgDJ-1 required for activation of CDPK1 could be a reflection of the low *K*_*d*_ of the interaction. Typical “biologically relevant” protein-protein interactions have a *K*_*d*_ in the low micromolar range, which would be consistent with our *in vitro* data for CDPK1 kinase activity. Such a low-affinity and possibly transient interaction may also explain why we were unable to isolate the complex from cells expressing native levels of tagged versions of either target. In further agreement with the presence of a potentially low-affinity complex that is regulated by multiple factors, our attempts to generate a crystal structure of the DJ-1/CDPK1 complex failed due to the fact that it does not appear to be stable enough to allow its isolation in sufficient purity for structural studies. Though we are confident that our data support this model, the stabilization and capture of the TgDJ-1/CDPK1 complex from within the cellular environment are top priorities for future studies.

In addition to the role of calcium and H_2_O_2_, the formation of the DJ-1/CDPK1 complex exhibited binary dependence upon the phosphostate of the kinase. The identification of a conserved arginine dyad as the primary site for recognition of phosphate is particularly important as it provided us with the ability to alter the interaction of CDPK with DJ-1 by mutation of these residues. Given the similarity between TgDJ-1 and other DJ-1 orthologues, it seems reasonable to speculate that the arginine dyad coordinates the tetragonal phosphate anion, with each residue contributing to the coordination. Therefore, the expectation would be that a single arginine would not be able to correctly coordinate the phosphate, and this is what we observed for the single-point mutant. However, mutation of both residues of the dyad to alanine should dramatically increase the overall space where the phosphate binds, and also remove the charges in the pocket. This could allow binding of CDPK1 regardless of its phosphorylation state, as we observed in our studies. Furthermore, the TgDJ-1/CDPK1 interaction is observed only under reducing conditions, with oxidation necessary for dissociation. Our crystal structure of TgDJ-1 indicates that, when oxidized, Cys104 may electrostatically clash with the anion coordinated by the Arg dyad, which may function to destabilize the interaction. Though it is generally highly conserved, we were surprised to note that the arginine dyad is not conserved in the closely related (but evolutionarily divergent) apicomplexan parasite, *Plasmodium* spp. This could mean that the interaction of DJ-1 with the equivalent kinase in *Plasmodium* does not occur, and that alternative regulatory mechanisms may have evolved to tune the activity of the kinase primarily responsible for coordinating microneme secretion in this parasite.

We initially found it somewhat surprising that, in our biochemical assays, the double-arginine mutant TgDJ-1 associated with CDPK1 independently of its phosphorylation state and yet failed to complement the loss of WT TgDJ-1 function in cells. Our results may be explained by the fact that TgDJ-1 is a dimer, with the arginine dyad consisting of residues contributed by both monomers of the dimer. Thus, by expressing a mutant monomer in cells that also express the WT TgDJ-1, it is possible that we generated mixed heterodimers in which one of the two binding sites contains the drug-sensitive Cys127 that has the WT arginine residues and the other contains the insensitive C127A mutation carrying the arginine mutations. This would result in a drug-insensitive complex containing only a single arginine site mutation which, like the TgDJ-1^C127A/R47A^ protein, would not productively form complexes with CDPK1. However, it should be noted that the overexpression of the mutant alleles would likely make this heterodimer scenario rare. Alternatively, the change in the nature of binding of the double-arginine mutant (i.e., to a non-phospho-dependent mode) could prevent it from supporting normal microneme secretion because a key part of the regulatory mechanism that controls which phosphorylated form of CDPK1 binds would be lost.

The conservation of the arginine dyad across diverse DJ-1 orthologues draws attention to the possibility that these residues have a conserved function in other organisms, including humans. For example, the human DJ-1 protein has been shown to interact with a wide variety of proteins such as DJBP (24) and, intriguingly, apoptosis signal-regulating kinase 1 (34). Given that the interaction of TgDJ-1 with CDPK1 directly affects kinase activity and the high sequence and structure conservation (35% identity for *Toxplasma gondii* versus *Homo sapiens*; root mean square deviation [RMSD] = 1.45), it would be worthwhile to determine if the human orthologue can similarly modulate the activity of client proteins such as ASK1.

In conclusion, we have demonstrated that the rather enigmatic protein TgDJ-1 forms a specific and highly regulated association with CDPK1. We show that TgDJ-1 complexes with CDPK1 and synergizes with calcium in order to potentiate CDPK1 activity. Our data suggest that this association is necessary to maintain normal microneme secretion and that genetic or chemical manipulations that destabilize or block the interaction consequently inhibit exocytosis of these important organelles. Furthermore, our findings suggest that intracellular signals (calcium, redox) that regulate complex stability may function in tandem to further fine-tune this process. Taken together, our results provide a mechanistic understanding of TgDJ-1 function in *T. gondii* microneme exocytosis and identify a previously unknown role for TgDJ-1 as a kinase-regulatory scaffold. Importantly, these results provide a foundation for studies that may shed light on how this protein functions in humans, where it plays an important role in the pathology of diseases such as Parkinson’s disease.

## MATERIALS AND METHODS

### Parasite strains.

The parasite strains used in this study are listed in Table 1.

**TABLE 1:** Parasite strain reference table

Strain name	Genetic background	Promoter	Expression product
*T. gondii* ΔTg*dj-1*	RHΔ*ku80*	NA^*a*^	NA
*T. gondii* ΔTg*dj-1*^WTcomp^	RHΔ*ku80*ΔTg*dj-1*	*GRA1*	FLAG-TgDJ-1^WT^
*T. gondii* CDPK1-HA	RHΔ*HXGPRT*	*GRA1*	CDPK1-HA
*T. gondii* TgDJ-1/TgDJ-1^C127A^	RHΔ*HXGPRT*	*GRA1*	FLAG-TgDJ-1^C127A^
*T. gondii* TgDJ-1/TgDJ-1^C127A/C104A^	RHΔ*HXGPRT*	*GRA1*	FLAG-TgDJ-1^C127A/C104A^
*T. gondii* TgDJ-1/TgDJ-1^C127A/R47A^	RHΔ*HXGPRT*	*GRA1*	FLAG-TgDJ-1^C127A/R47A^
*T. gondii* TgDJ-1/TgDJ-1^C127A/R27A/R47A^	RHΔ*HXGPRT*	*GRA1*	FLAG-TgDJ-1^C127A/R27A/R47A^

a NA, not applicable.

### Compound synthesis.

WRR-086 was synthesized as previously described (18).

### Parasite and host cell maintenance.

Type I *T. gondii* strain RH was maintained by passage through confluent monolayers of human foreskin fibroblasts (HFFs). Host cells were cultured as previously described (35). Parasites were harvested for use in assays by either syringe lysis of infected HFF monolayers or collection of parasites from culture supernatant after spontaneous lysis of the monolayer.

### Parasite transfection and isolation of single-cell clones.

Transgenic parasite strains were made by electroporating the *T. gondii* strain RHΔku80 (36) with 15 μg of linearized plasmid encoding the construct of interest and selecting for mycophenolic acid (MPA)-xanthine- or 5-fluorodeoxyuridine (FUdR)-resistant parasites, as described previously (37, 38). Clonal parasites were selected by limiting dilution. Integration was verified using a locus-specific PCR. Details of all strains used can be found in Table 1.

### Transfection constructs.

Plasmids for the construction of vectors described were obtained from the Boothroyd laboratory. These include pTKO2 (used to generate the ΔTg*DJ-1* parasite strain), pUPRT (used to complement ΔTg*DJ-1* parasites), and pGRA (used to generate the merodiploid strains). For the generation of the ΔTg*DJ-1* parasite strain, the T1 sequence was amplified using primers 5′-TGTTTCATGACGTACCCGACACAGCAC-3′ and 5′-GCGACGGGCTGCGGTAGG-3′ and the T2 sequence was amplified using primers 5′-AAGCTTAAGCTTGAGTCGTTACAGCCTAATGAAGCG-3′ and 5′-GGGCCCCAAGTCCGAATCTGCCTAACTCC-3′.

### Mutagenesis.

In all cases, mutagenesis was performed by site-directed mutagenesis (Stratagene) or using the Phusion site-directed mutagenesis technique (Thermo).

### Cell assays.

For all of the cell assays, compound treatments were performed as follows: intracellular parasites were released from heavily infected host cell monolayers by syringe lysis. Hanks balanced salt solution (HBSS)-washed parasites were incubated with hydrogen peroxide or compound for 15 min at 37°C at the concentration described in the text (unless otherwise stated) before being used for the appropriate cell assay.

### Plaque assays.

Toxoplasma plaque formation was assayed as described previously (39).

### Microneme secretion assays.

Microneme secretion assays quantifying the release of MIC2 into the extracellular medium were performed as described previously (40), with modifications described previously (35). For quantification, the following approach was used: mean pixel intensities for GRA7 loading control bands were measured in ImageJ. Band intensities were initially normalized to the background intensity measurements of a sample region from the same lane on the blot. GRA7 background subtracted intensity readings were then compared to the readings from the control lane (e.g., zero time point, dimethyl sulfoxide [DMSO] treatment). This generated the loading adjustment factor to normalize for loading against constitutively secreted GRA7. Mean pixel intensities for MIC2 bands were then measured in ImageJ. Band intensities were initially normalized to the background of a sample region from the same lane. Background subtracted intensity readings were then multiplied by the adjustment factor determined above to adjust for loading. Mean values are presented for 3 replicates.

### Gliding motility and attachment/invasion assays.

2D motility and the attachment/invasion of host cells were assayed as described previously (35).

### Biochemical fractionation of TgDJ-1.

RHΔ*ku80* tachyzoites (50 × 10^8^) were washed with HBSS, pelleted, and snap-frozen. The cell pellet was thawed and resuspended in 200 µl of hypotonic lysis buffer (50 mM HEPES, pH 7) and incubated on ice for 30 min. Samples were then spun at 100 × *g* for 1 h. The supernatant was separated as the “soluble” fraction, and the pellet was resuspended in 200 µl of phosphate-buffered saline (PBS) as the “insoluble” fraction. Fractions were solubilized with equivalent amounts of reducing Laemmli SDS-PAGE sample buffer, and equivalent volumes were analyzed by SDS-PAGE and Western blotting.

### Immunofluorescence assays.

Immunofluorescence assays were performed as described previously (35) with the antibodies indicated in relevant figure legends.

### Preparation of cell extracts.

Cell pellets of HBSS-washed extracellular tachyzoites were obtained for the relevant parasite strains. Pellets were thawed in lysis buffer (50 mM Tris [pH 7.4], 150 mM NaCl, 0.5% NP-40) and incubated on ice for 30 min. Extracts were clarified by centrifugation at 14,000 rpm and 4°C. Supernatants were separated and retained as the cell extracts. The protein concentration of extracts was quantified by bicinchoninic acid (BCA) assay to ensure that equivalent amounts of protein were available for use in the respective comparative assays.

### Immunoprecipitations.

Immunoprecipitations (IPs) were performed as follows (unless otherwise specified in the text). Typically, 200 µg cell extract protein was added to 200 µl of IP buffer (50 mM Tris [pH 7.4], 150 mM NaCl, 0.5% NP-40). Anti-HA beads (Pierce) (40 μl) were then added to the sample, and the IPs were performed according to the instructions of the manufacturer. For IPs using conditioned extracts and recombinant FLAG-tagged TgDJ-1, the samples were adjusted to be reducing and calcium depleted (2 mM DTT, 5 mM EGTA), oxidizing and calcium depleted (H_2_O_2_ [at concentrations specified in the figure legend or text], 5 mM EGTA), reducing and calcium rich (2 mM DTT, 5 mM CaCl_2_), or oxidizing and calcium rich (H_2_O_2_ [at concentrations specified in the figure legend or text], 5 mM CaCl_2_). Once adjusted, IP samples containing the extracts were incubated for 15 min on ice. FLAG-TgDJ-1 (5 μg) was then added to each, and the samples were incubated for a further 5 min on ice before the addition of 40 µl of anti-HA agarose beads (Pierce), with the IP performed according to the instructions of the manufacturer. Specific extract conditions were maintained for all wash steps unless otherwise stated.

### Protein expression and purification.

The *Toxoplasma gondii* DJ-1 gene (TgDJ-1) (18) was PCR amplified using primers that introduced a 5′ NdeI restriction site and a 3′ BamHI site. The amplified insertion was digested with NdeI and BamHI and cloned between these restriction sites in the bacterial expression vector pET15b (Novagen). The C104S and C127S point mutations were made using standard site-directed mutagenesis with mutagenic primers. The constructs were transformed into chemically competent *E. coli* strain BL21(DE3) (Novagen Merck, Darmstadt, Germany,) for protein expression. The recombinant protein has a thrombin-cleavable N-terminal hexahistidine tag to facilitate purification using Ni^2+^ metal affinity chromatography. Protein expression and purification were performed as described previously (41), except that all buffers contained 2 mM DTT. The absorbance of the purified sample was measured at 280 nm in DTT-free buffer and converted to a protein concentration using a calculated extinction coefficient for TgDJ-1 at 280 nm (ε_280_) of 7,825 M^−1^ cm^−1^ (Expasy) and a molecular mass of 29,296 Da. The purified protein was concentrated to 20 mg/ml in storage buffer (25 mM HEPES [pH 7.5], 100 mM KCl, 2 mM DTT) using a stirred pressure cell concentrator with a 10-kDa molecular mass cutoff. The purified protein ran as a single band on an overloaded SDS-PAGE gel stained with Biosafe Coomassie blue (Bio-Rad, Hercules, CA). The concentrated protein was divided into aliquots, rapidly frozen in liquid nitrogen, and stored at −80°C.

For GST-CDPK1 constructs, CDPK1 was amplified from cDNA and cloned into pGEX-6P1 for expression with an N-terminal GST tag. Protein was expressed as described above. For preparations of dephosphorylated GST-CDPK1, the vector was coexpressed with a construct for the expression of lambda phosphatase. Following expression, GST-tagged recombinant proteins were purified using glutathione-Sepharose beads. Following binding, beads were washed extensively with lysis buffer with 5 mM EGTA and 2 mM DTT and then stored at −20°C as a 50% slurry in lysis buffer with 5 mM EGTA and 2 mM DTT and 50% glycerol.

### GST pulldown.

Glutathione beads (10 μl) coated with GST, GST-CDPK1, or GST-CDPK1 coexpressed with lambda phosphatase were added to 200 µl of pulldown buffer (50 mM Tris [pH 7.4], 150 mM NaCl, 5 mM EGTA, 2 mM DTT) and 1 µg of recombinant TgDJ-1 (wild-type or mutants as specified in the text or the figure legends). Samples were incubated with agitation performed at 4°C for 1 h. Beads were then washed three times with 500 µl of pulldown buffer, and associated proteins were solubilized with equal volumes of reducing Laemli sample buffer and submitted for analysis by SDS-PAGE, Coomassie staining, and Western blotting.

### Antibodies.

Polyclonal murine antibodies were raised against TgDJ-1 as previously described (31). Polyclonal murine antibodies against CDPK1 were generated in a previous study (31).

### Profiling of TgDJ-1 cysteine reactivity.

*E. coli* lysates overexpressing TgDJ-1 were diluted to a 2 mg protein/ml solution in PBS. Each sample (2× 0.5-ml aliquots) was treated with an iodoacetamide probe at a concentration of either 10 or 100 µM using 5 µl of a 1 mM or 10 mM stock in DMSO. The labeling reaction mixtures were incubated at room temperature for 1 h. Click chemistry was performed by the addition of a 150 µM concentration of either a light-tobacco etch virus (TEV) tag (for 10 µM iodoacetamide [IA] samples) or a heavy TEV tag (for 100 µM IA samples) (15 µl of a 5 mM stock) as described in reference 19. Samples were allowed to react at room temperature for 1 h. After the click chemistry step, the light-tag- and heavy-tag-labeled samples were mixed together and centrifuged (5,900 × *g*, 4 min, 4°C) to pellet the precipitated proteins. The pellets were washed twice in cold MeOH, after which the pellet was solubilized in PBS containing 1.2% SDS via sonication and heating (5 min and 80°C). Upon resolubilization, the proteome sample was subjected to streptavidin enrichment and on-bead trypsin and TEV digestion as previously described (19). The resulting probe-labeled peptides obtained after TEV digestion were analyzed on a linear trap quadrupole (LTQ)-Orbitrap Discovery mass spectrometer (Thermo, Fisher) using the Mudpit protocol as previously reported (19). Peptide identification was achieved using the SEQUEST algorithm and quantified using CIMAGE as reported previously (19).

### Spectrophotometric cysteine p*K*_a_ determination.

The p*K*_a_ value of the reactive cysteine residue (Cys104) in TgDJ-1 was measured by monitoring the absorption of the thiolate anion at 240 nm as a function of pH (20, 21). A triple buffer (10 mM boric acid, 10 mM sodium citrate, 10 mM sodium phosphate) was prepared, and TgDJ-1 protein was added to reach a final concentration of 10 μM. The pH of each sample was changed by the addition of 0.5- to 1.0-μl aliquots of 0.5 N or 5.0 N NaOH. After each addition of NaOH, the absorbance at 240 and 280 nm was measured using a Cary 50 spectrophotometer (Varian, Inc., Palo Alto, CA). The *A*_280_ value was used to normalize the *A*_240_ measurement to the protein concentration in the cuvette. After spectrophotometric measurement, the pH of each sample was measured using an Orion micro-pH electrode (Thermo Fisher, Waltham, MA, USA) that had been calibrated at the beginning of the experiment. Measured absorbance values were converted to an extinction coefficient at 240 nm (ε_240_) using a calculated ε_280_ value for TgDJ-1 of 7,825 M^−1^ cm^−1^ (Expasy). The experiment was performed in triplicate for wild-type protein and once for the C127S and C104S mutants. The data were fitted to a modified version of the Henderson-Hasselbalch equation (22) in Prism (GraphPad Software, Inc.). The reported p*K*_a_ values and associated errors are derived from the fit procedure.

### Protein expression and purification for crystallography.

All recombinant protein was expressed in *E. coli* Rosetta2(DE3) (EMD Biosciences). N-terminally His_6_-tagged TgDJ-1 was purified on nickel-nitrilotriacetic acid (Ni-NTA) resin (Qiagen), and the His_6_ tag was removed by overnight cleavage using thrombin (Hematologic Technologies) at 4°C. All proteins were further purified by anion exchange and gel filtration chromatography. After elution from Ni-NTA, TgDJ-1 was kept in fresh 10 mM DTT for all subsequent steps.

### Crystallization conditions.

High-quality crystals of TgDJ-1 grew from a 1:1 mixture of protein (5 mg/ml)–10 mM HEPES (pH 7.0)–100 mM NaCl–10 mM DTT and 1.5 M ammonium citrate tribasic (Hampton Research). Crystals were flash frozen in mother liquor for data collection.

### Data collection, structure determination, and refinement.

The diffraction data were collected at beamline 11.1 of SSRL (the Stanford Synchrotron Radiation Laboratory) at a wavelength of 0.979 Å and a temperature of 100 K. Indexing, integration, and scaling of the diffraction data were performed using the XDS suite (42). Initial phases were obtained by molecular replacement using Phaser (43) and searching with a homology model of TgDJ-1 created from HsDJ-1 with Modeller (44). Manual rebuilding in Coot (45) and refinement in refmac (46, 47) led to a final 2.08-Å structure which was deposited in the Protein Data Bank. The structure showed good stereochemistry (97.5% favored) from Ramachandran plots as validated by the program MOLPROBITY (48).

### Structural figure generation.

All structural images were created using PyMOL 1.7.0 (Schrödinger, LLC).

### *In vitro* kinase assays.

Kinase assays were performed using a kinase Glo kit (Promega) and were optimized according to the instructions of the manufacturer with the following modifications. A standard assay would include two incubation steps: an initial 30-min incubation at 37°C of a reaction mixture typically containing recombinant CDPK1 kinase, 50 nM CaCl_2_, kinase buffer (50 mM Tris [pH 7.4], 10 mM MgCl_2_, 2 mM DTT), and TgDJ-1 (unless otherwise stated). Following this initial incubation, 10 µM ATP, 10 µM syntide, and 500 µM CaCl_2_ were added (unless otherwise stated) and the reaction mixture was incubated for a further 30 min at 37°C. Assays were then processed according to the instructions of the manufacturer.

### Accession number(s).

The 2.08-Å structure determined in this work was deposited in the Protein Data Bank (PDB accession no. 4XLL).
