# Supplementary figures and images for: Toxoplasma DJ-1 Regulates Organelle Secretion by a Direct Interaction with Calcium-Dependent Protein Kinase 1
Source: mBio. 2017 Feb 28;8(1):e02189-16. doi: 10.1128/mBio.02189-16 (PMC5347346; doi:10.1128/mBio.02189-16)

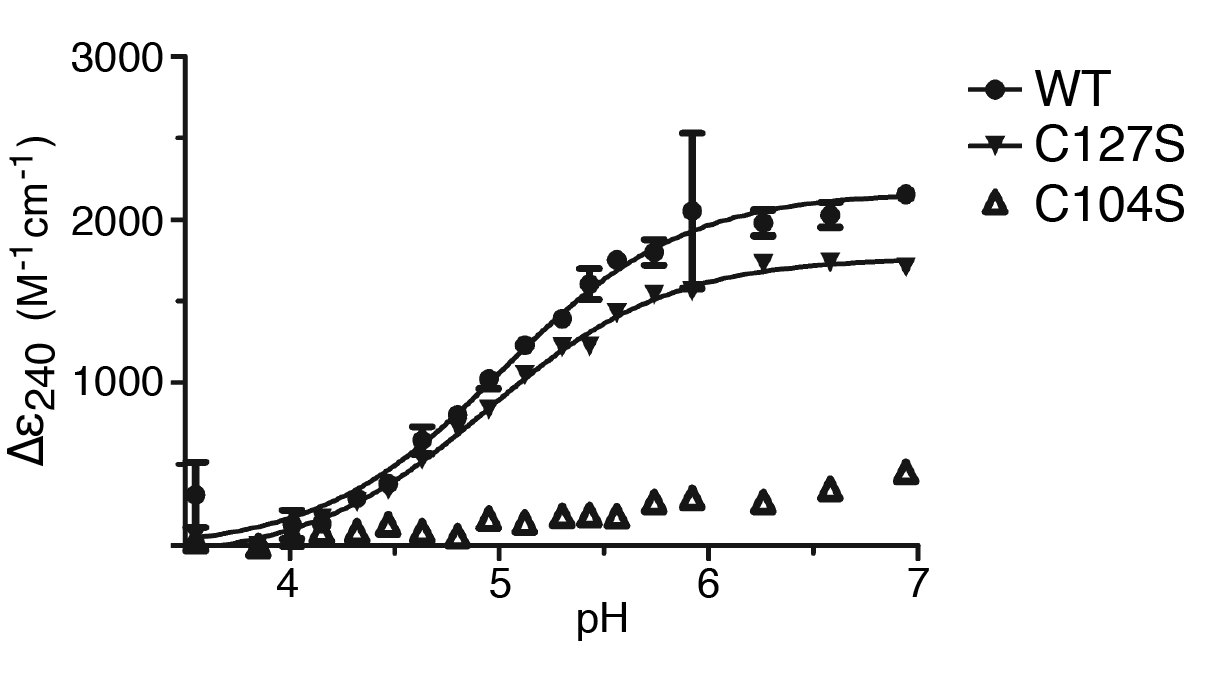

Supplement: FIG S1 [file mbo001173207sf1.tif]

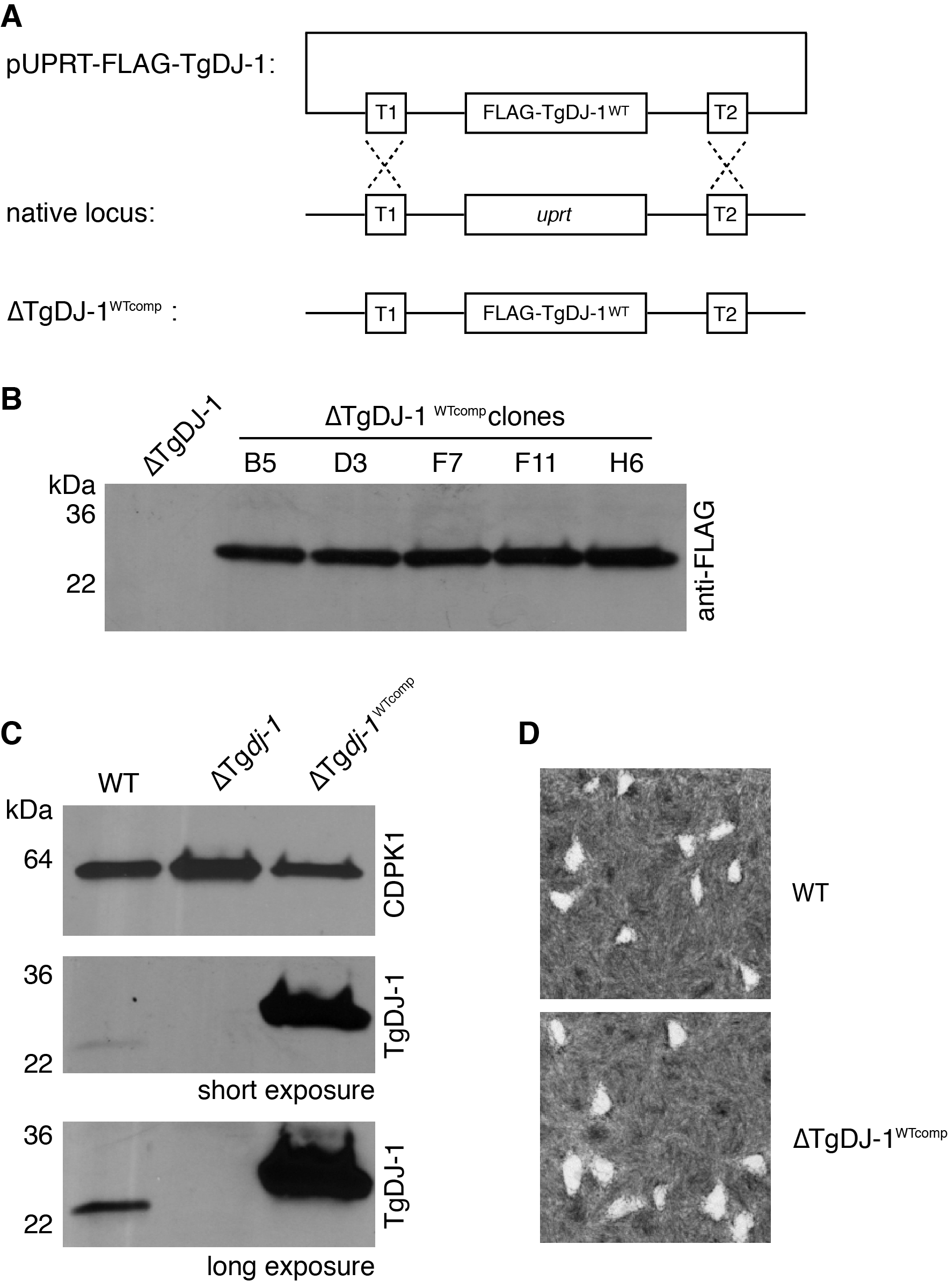

Supplement: FIG S3 [file mbo001173207sf3.tif]

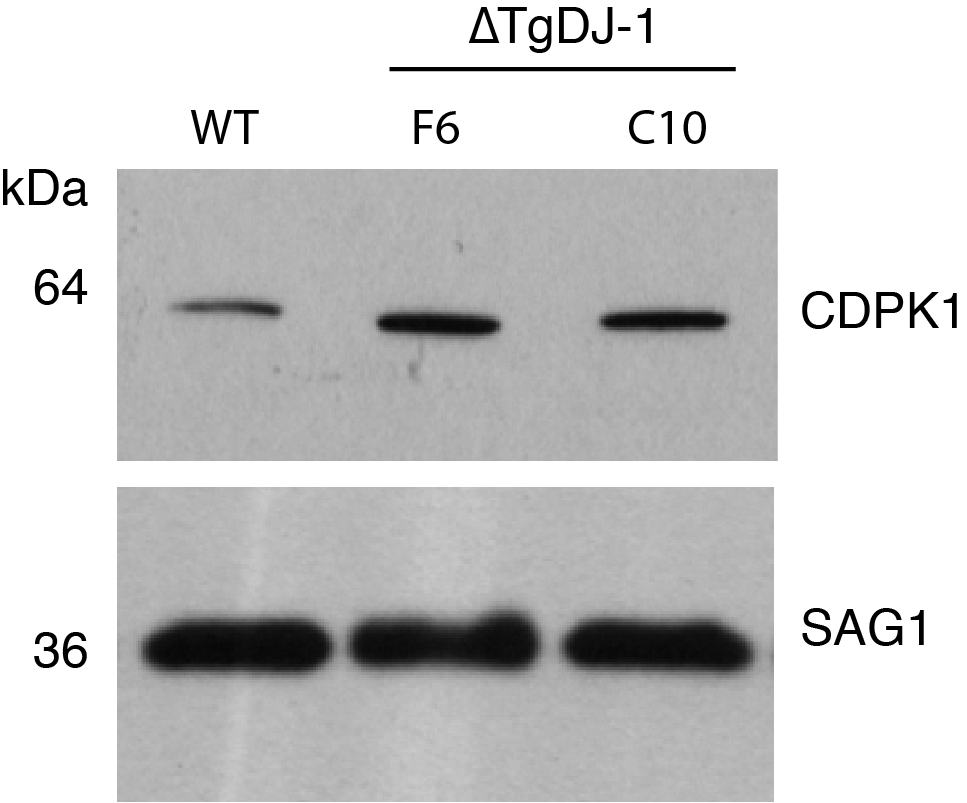

Supplement: FIG S4 [file mbo001173207sf4.tif]

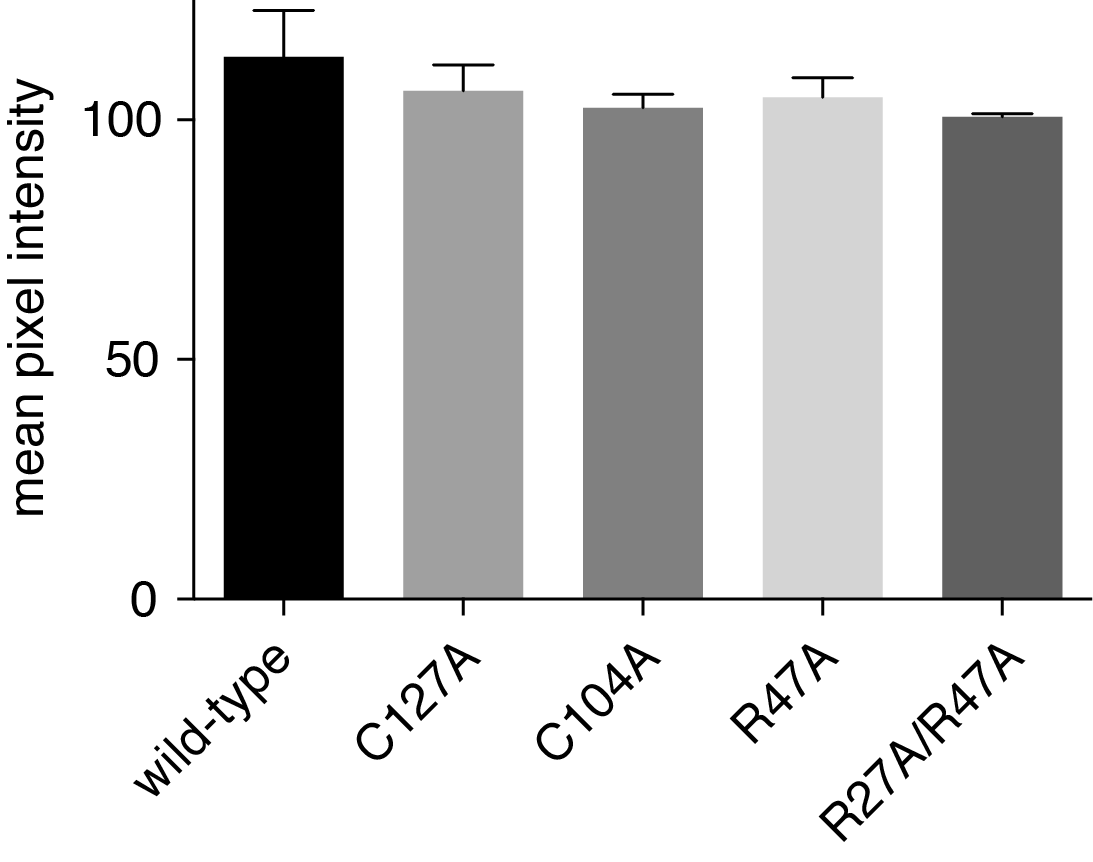

Supplement: FIG S5 [file mbo001173207sf5.tif]
