# Supplementary material for: Toxoplasma DJ-1 Regulates Organelle Secretion by a Direct Interaction with Calcium-Dependent Protein Kinase 1
Source: mBio. 2017 Feb 28;8(1):e02189-16. doi: 10.1128/mBio.02189-16 (PMC5347346; doi:10.1128/mBio.02189-16)
Supplement: TABLE S1 [file mbo001173207st1.docx]

**Table S1.** Data for the post-hoc pairwise comparison of basal microneme secretion in Figure 5B performed using Tukey’s multiple comparison test. ns, not significant. *, P ≤0.05. **, P ≤0.01. ***, P ≤0.001. ****, P ≤0.0001.

| Timepoint | Pairwise comparison | Significance |
| --- | --- | --- |
| 15 | WT *vs.* ∆Tg*dj-1* | * |
| 15 | WT *vs.* ∆Tg*dj-1*^WTcomp^ | ns |
| 15 | ∆Tg*dj-1 vs.* ∆Tg*dj-1*^WTcomp^ | * |
| 30 | WT *vs.* ∆Tg*dj-1* | *** |
| 30 | WT *vs.* ∆Tg*dj-1*^WTcomp^ | **** |
| 30 | ∆Tg*dj-1 vs.* ∆Tg*dj-1*^WTcomp^ | **** |
| 60 | WT *vs.* ∆Tg*dj-1* | ns |
| 60 | WT *vs.* ∆Tg*dj-1*^WTcomp^ | **** |
| 60 | ∆Tg*dj-1 vs.* ∆Tg*dj-1*^WTcomp^ | **** |
