# Supplementary material for: Toxoplasma DJ-1 Regulates Organelle Secretion by a Direct Interaction with Calcium-Dependent Protein Kinase 1
Source: mBio. 2017 Feb 28;8(1):e02189-16. doi: 10.1128/mBio.02189-16 (PMC5347346; doi:10.1128/mBio.02189-16)
Supplement: TABLE S2 [file mbo001173207st2.docx]

**Table S2.** Data for the post-hoc pairwise comparison of induced microneme secretion in Figure 5D performed using Tukey’s multiple comparison test. ns, not significant. *, P ≤0.05. **, P ≤0.01. ***, P ≤0.001. ****, P ≤0.0001.

| % EtOH | Pairwise comparison | Significance |
| --- | --- | --- |
| 0 | WT *vs.* ∆Tg*dj-1* | ns |
| 0 | WT *vs.* ∆Tg*dj-1*^WTcomp^ | ns |
| 0 | ∆Tg*dj-1 vs.* ∆Tg*dj-1*^WTcomp^ | ns |
| 0.1 | WT *vs.* ∆Tg*dj-1* | ** |
| 0.1 | WT *vs.* ∆Tg*dj-1*^WTcomp^ | ns |
| 0.1 | ∆Tg*dj-1 vs.* ∆Tg*dj-1*^WTcomp^ | *** |
| 1 | WT *vs.* ∆Tg*dj-1* | **** |
| 1 | WT *vs.* ∆Tg*dj-1*^WTcomp^ | *** |
| 1 | ∆Tg*dj-1 vs.* ∆Tg*dj-1*^WTcomp^ | **** |
