# Supplementary material for: Toxoplasma DJ-1 Regulates Organelle Secretion by a Direct Interaction with Calcium-Dependent Protein Kinase 1
Source: mBio. 2017 Feb 28;8(1):e02189-16. doi: 10.1128/mBio.02189-16 (PMC5347346; doi:10.1128/mBio.02189-16)
Supplement: TABLE S3 [file mbo001173207st3.docx]

**Table S3.** Data for the post-hoc pairwise comparison of induced microneme secretion in the presence of titration of WRR-086 in Figure 6E, performed using Tukey’s multiple comparison test. ns, not significant. *, P ≤0.05. **, P ≤0.01. ***, P ≤0.001. ****, P ≤0.0001.

| µM WRR-086 | Pairwise comparison | Significance |
| --- | --- | --- |
| 0 | wild-type vs. C127A | ns |
| 0 | wild-type vs. C127A/C104A | ns |
| 0 | wild-type vs. C127A/R47A | ns |
| 0 | wild-type vs. C127A/R27A/R47A | ns |
| 0 | C127A vs. C127A/C104A | ns |
| 0 | C127A vs. C127A/R47A | ns |
| 0 | C127A vs. C127A/R27A/R47A | ns |
| 0 | C127A/C104A vs. C127A/R47A | ns |
| 0 | C127A/C104A vs. C127A/R27A/R47A | ns |
| 0 | C127A/R47A vs. C127A/R27A/R47A | ns |
| 1 | wild-type vs. C127A | ns |
| 1 | wild-type vs. C127A/C104A | ns |
| 1 | wild-type vs. C127A/R47A | ns |
| 1 | wild-type vs. C127A/R27A/R47A | * |
| 1 | C127A vs. C127A/C104A | ns |
| 1 | C127A vs. C127A/R47A | ns |
| 1 | C127A vs. C127A/R27A/R47A | ns |
| 1 | C127A/C104A vs. C127A/R47A | ns |
| 1 | C127A/C104A vs. C127A/R27A/R47A | * |
| 1 | C127A/R47A vs. C127A/R27A/R47A | ns |
| 5 | wild-type vs. C127A | **** |
| 5 | wild-type vs. C127A/C104A | ** |
| 5 | wild-type vs. C127A/R47A | ns |
| 5 | wild-type vs. C127A/R27A/R47A | * |
| 5 | C127A vs. C127A/C104A | ns |
| 5 | C127A vs. C127A/R47A | **** |
| 5 | C127A vs. C127A/R27A/R47A | ns |
| 5 | C127A/C104A vs. C127A/R47A | * |
| 5 | C127A/C104A vs. C127A/R27A/R47A | ns |
| 5 | C127A/R47A vs. C127A/R27A/R47A | * |
| 10 | wild-type vs. C127A | **** |
| 10 | wild-type vs. C127A/C104A | ns |
| 10 | wild-type vs. C127A/R47A | ns |
| 10 | wild-type vs. C127A/R27A/R47A | ns |
| 10 | C127A vs. C127A/C104A | *** |
| 10 | C127A vs. C127A/R47A | **** |
| 10 | C127A vs. C127A/R27A/R47A | **** |
| 10 | C127A/C104A vs. C127A/R47A | ns |
| 10 | C127A/C104A vs. C127A/R27A/R47A | ns |
| 10 | C127A/R47A vs. C127A/R27A/R47A | ns |
