# Supplementary material for: Toxoplasma DJ-1 Regulates Organelle Secretion by a Direct Interaction with Calcium-Dependent Protein Kinase 1
Source: mBio. 2017 Feb 28;8(1):e02189-16. doi: 10.1128/mBio.02189-16 (PMC5347346; doi:10.1128/mBio.02189-16)
Supplement: TABLE S4 [file mbo001173207st4.docx]

**Table S4.** **Data collection and refinement statistics**

|  | TgDJ-1, oxidized (4XLL) |
| --- | --- |
| **Data collection** |  |
| Space group | P 2 1 1 |
| Cell dimensions |  |
| *a*, *b*, *c* (Å) | 38.78, 55.18, 75.25 |
| α, β, γ (°) | 90, 93.919, 90 |
| Resolution (Å) | 2.08 |
| *R*_sym_ | 9.2 (33.8) |
| *I* / σ*I* | 16.96 (5.25) |
| Completeness (%) | 97.2 (90.3) |
| Redundancy | 6.85 (6.30) |
|  |  |
| **Refinement** |  |
| Resolution (Å) | 2.08 |
| No. reflections | 16108 |
| *R*_work_ / *R*_free_ | 0.16856 / 0.21876 |
| No. atoms |  |
| Protein | 2745 |
| Water | 245 |
| *B*-factors |  |
| Protein | 22.0 |
| Water | 28.20 |
| R.m.s. deviations |  |
| Bond lengths (Å) | 0.015 |
| Bond angles (°) | 1.7 |
